# Safety and efficacy of etelcalcetide, an intravenous calcimimetic, for up to 52 weeks in hemodialysis patients with secondary hyperparathyroidism: results of a post-marketing surveillance in Japan

**DOI:** 10.1007/s10157-020-01936-2

**Published:** 2020-08-20

**Authors:** Keitaro Yokoyama, Masafumi Fukagawa, Takashi Shigematsu, Takashi Akiba, Ken Yoshikawa, Akira Tsuchiya, Misato Kuwabara, Tadao Akizawa

**Affiliations:** 1 Harumi Triton Clinic, The Jikei University Hospital, 1-8-8 Harumi, Chuo-ku, Tokyo, 104-0053 Japan; 2 Division of Nephrology, Endocrinology and Metabolism, Tokai University School of Medicine, Isehara, Japan; 3 Department of Nephrology, Wakayama Medical University, Wakayama, Japan; 4 Tokyo Next Nephrology and Dialysis Clinic, Tokyo, Japan; 5 Department of Pharmacovigilance, Drug Reliability Assurance, ONO Pharmaceutical Co., Ltd., Osaka, Japan; 6 Division of Nephrology, Department of Medicine, Showa University School of Medicine, Tokyo, Japan

**Keywords:** Etelcalcetide, Calcimimetics, Post-marketing surveillance, Hemodialysis, Secondary hyperparathyroidism, Clinical practice

## Abstract

**Background:**

Etelcalcetide is a second-generation calcimimetic for the management of secondary hyperparathyroidism (SHPT) in patients on dialysis. We performed a post-marketing surveillance (PMS) to obtain information on the safety and efficacy of etelcalcetide in clinical practice in Japan.

**Methods:**

This PMS enrolled SHPT patients who started initial treatment with etelcalcetide between April 1, 2017 and February 28, 2018 in Japan. Safety [adverse drug reactions (ADRs)] and efficacy [serum intact parathyroid hormone (iPTH), corrected calcium (cCa), phosphorous (P), and alkaline phosphatase (ALP)] were recorded for up to 52 weeks or until treatment discontinuation. Treatment decisions were at the physician’s discretion.

**Results:**

Of 1226 patients enrolled across 282 centers, safety and efficacy data were available for 1195 and 1192, respectively, while 933 continued treatment to Week 52. The starting dose was 5 mg in 82.0% of patients. There were 218 ADRs in 169 patients (14.1%). Metabolism and nutrition disorders (8.8%), adverse laboratory test results (1.8%), and gastrointestinal disorders (1.6%) were the most frequent classes of ADRs. Hypocalcemia-related ADRs occurred in 104 patients (8.7%). The percentage of patients with iPTH levels within the target range (60–240 pg/mL) steadily increased from 19.5% at Week 0 to 64.1% at Week 52 or last dose. cCa, P, and ALP levels remained well controlled.

**Conclusion:**

This was the first real-world, large-scale, long-term observational PMS of etelcalcetide in Japan. We did not observe any new safety concerns. Etelcalcetide was associated with clinically relevant improvements in serum iPTH and maintenance of serum cCa, P, and ALP levels.

**Electronic supplementary material:**

The online version of this article (10.1007/s10157-020-01936-2) contains supplementary material, which is available to authorized users.

## Introduction

Secondary hyperparathyroidism (SHPT) is a potential complication of hemodialysis or hemodiafiltration in patients with chronic kidney disease. It is characterized by the excessive release of parathyroid hormone (PTH) from the parathyroid glands in response to a combination of elevated phosphorus (P), decreased calcium (Ca), and decreased 1,25-dihydroxyvitamin D levels, resulting in parathyroid hyperplasia [1, 2].

The 2017 Kidney Disease: Improving Global Outcomes (KDIGO) guidelines suggest the use of calcimimetics, calcitriol, vitamin D analogs, or a combination of calcimimetics with calcitriol or vitamin D analogs for patients on dialysis requiring PTH-lowering therapy. The guidelines also suggest that serum intact PTH (iPTH) levels should be maintained within a range of about two to nine times the assay’s upper limit of normal [3]. The Japanese Society for Dialysis Therapy [4] guidelines suggest that serum P and Ca levels should be controlled first, followed by the management of serum PTH levels, with a suggested target range of 60–240 pg/mL, using appropriate medical therapy (serum P/Ca management, vitamin D receptor activators, and/or cinacalcet hydrochloride).

Etelcalcetide is an intravenous calcimimetic agent that activates the calcium-sensing receptor and inhibits the release of PTH from the parathyroid glands, and hence decreases serum Ca levels [5, 6]. Etelcalcetide has been approved in several countries for the treatment of SHPT in patients on hemodialysis. Clinical trials used for the approval of novel drugs remain an important aspect of drug development and approval. However, they may not fully reflect the reality of clinical practice or provide comprehensive insight into the safety of newly approved drugs when used in clinical settings. In particular, clinical trials often exclude some specific categories of patients. For etelcalcetide, the prior trials excluded patients whose last dose of cinacalcet was < 28 days before starting etelcalcetide, pretreatment serum iPTH was ≤ 240 pg/mL, or serum pretreatment corrected Ca (cCa) was < 8.4 mg/dL. Moreover, clinical trials are generally small, which may hinder the detection of important but rare adverse drug reactions (ADRs).

Post-marketing surveillance (PMS) can help to overcome these limitations and provide valuable information on the safety and efficacy of newly approved drugs in a large population of patients. Following the approval of etelcalcetide in Japan, a PMS was implemented to investigate its safety and efficacy for managing SHPT in a target population of ~ 1000 Japanese patients treated in actual clinical practice. Here, we report the final results of this PMS in patients treated with etelcalcetide for up to 52 weeks.

## Methods

### Ethics

This PMS was conducted as part of the mandatory actions for approval of etelcalcetide in Japan and complied with the Japanese ministerial ordinance on Good Post-marketing Study Practice. According to this ordinance, ethical approval of the participating medical institutions and patient consent is not required. The PMS was registered on the Japan Pharmaceutical Information Center database (JapicCTI-184074).

### Patients

Patients were to be enrolled in this Japanese PMS if they started treatment with etelcalcetide for the first time to manage SHPT during hemodialysis between April 1, 2017 and February 28, 2018. Patients were enrolled within 14 days of starting treatment of etelcalcetide and followed up for 52 weeks after starting treatment or until discontinuation.

### PMS design and treatments

All treatments were at the physician’s discretion with consideration of the approved labels for the prescribed products. In accordance with its package insert, etelcalcetide should be administered intravenously during dialysis at a starting dose of 5 mg three-times-weekly. Its dose can be adjusted across a range of 2.5–15 mg as deemed appropriate by the physician.

The package insert for etelcalcetide in Japan recommends maintaining serum PTH within a control target range, and that its dose can be reduced or its administration stopped if serum PTH falls below the control target level and/or serum cCa decreases to < 8.4 mg/dL. Although no specific serum iPTH level is recommended in the package insert, the Japanese Society for Dialysis Therapy [4] guidelines suggest a target range of 60–240 pg/mL.

The use and dosing of concomitant therapies were at the physician’s discretion. The type of dialysate, including the dialysate Ca concentration (2.5–3.0 mEq/L), was dependent on the dialysate used at the institution because most institutions in Japan use a central dialysate delivery system and the dialysate is rarely changed in individual patients.

### Data collection and endpoints

All data were recorded prospectively using an electronic data capture system. Information recorded at enrollment included the date on which etelcalcetide was started. This information was to be enrolled within 14 days of the start of etelcalcetide. The physicians also recorded patient characteristics, dialysis-related variables, history of cinacalcet use, etelcalcetide use, concomitant therapies, laboratory variables, and adverse events (AEs). Additionally, the physicians collected information on the use of concomitant therapies including active vitamin D preparations, Ca preparations, and phosphate-binders, and whether parathyroidectomy or percutaneous ethanol injection therapy were performed.

Safety was assessed in terms of the rates and types of ADRs, with a special focus on hypocalcemia, worsening of cardiac failure, QT prolongation, hypersensitivity, convulsions, and bone metabolism disorders. ADRs were classified as AEs that were regarded as having a causal relationship with etelcalcetide. ADRs and their severity (serious or non-serious) were primarily assessed by the attending physician. Any AEs that were not considered to be ADRs, and any ADRs that were not deemed to be serious ADRs by the physicians, could be re-classified as ADRs or as serious ADRs, as appropriate, by the sponsor.

Efficacy was evaluated in terms of changes in laboratory variables, focusing on serum iPTH, cCa, P, and alkaline phosphatase (ALP) levels. We also performed subgroup analyses to determine the proportion of patients who achieve serum iPTH levels within the target range.

The cCa levels were calculated by each institution using the following formula, and the values were collected by the sponsor:

cCa (mg/dL) = serum Ca (in mg/dL) − serum albumin (in g/dL) + 4.0.

### Statistical analyses

It was planned to enroll about 1200 patients, allowing safety analyses in about 1000 patients. This would enable us to detect unknown ADRs occurring at a frequency of ≥ 0.3% with a confidence level of ≥ 95%. Assuming each institution/department enrolled 5–10 patients, it was planned to enlist 120–240 participating medical institutions.

Descriptive statistics (number of patients, mean ± standard deviation, median [quartile 1–quartile 3], max, minimum) were determined for safety and efficacy variables. The percentages of patients with any ADR, with hypocalcemia, or who achieved serum iPTH within the target range (60–240 pg/mL) were determined together with 95% confidence intervals. These percentages were calculated for the overall population of patients and in prespecified subgroups of patients divided by baseline characteristics (sex, age, dry body weight, iPTH, cCa, P, ALP, history of dialysis, dialysis modality, dialysate Ca level, cinacalcet use within 28 days before starting etelcalcetide, and dose of last cinacalcet). Fisher’s exact test or the Mann–Whitney *U* test were performed as appropriate for subgroup analysis.

We performed logistic regression analyses using the appearance of hypocalcemia as an objective variable and possible factors affecting hypocalcemia as explanatory variables, which were selected using the forward–backward stepwise selection method, in the safety analysis set. We also performed logistic regression analyses using the proportion of patients who achieved serum iPTH within the target range as an objective variable, and selected possible explanatory factors using the forward–backward stepwise selection method, in the efficacy analysis set.

SAS version 9.4 (SAS Institute, Cary, NC, USA) and Microsoft Excel 2010 (Microsoft, Redmond, VA, USA) were used for all analyses.

## Results

### Patients and treatments

**Fig. 1:**
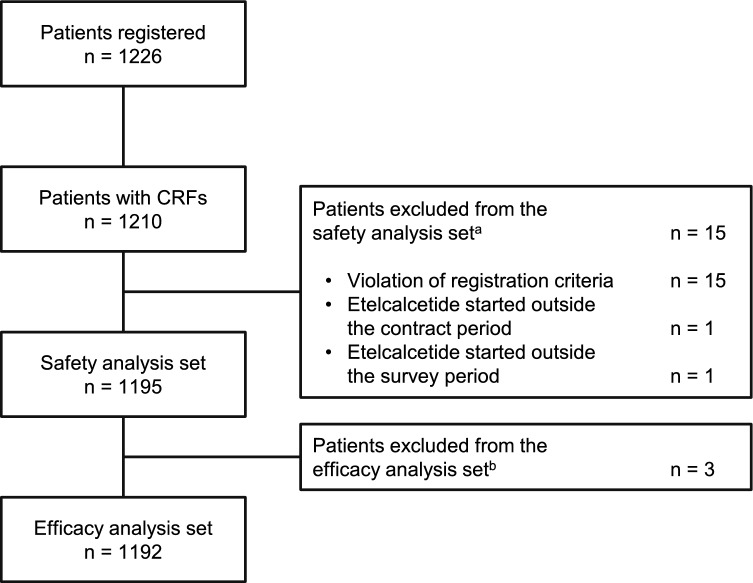
Patient disposition. *CRF* case report form. ^a^Multiple reasons may apply. ^b^Patients whose efficacy data were not measured for all four efficacy endpoints (serum corrected calcium, serum phosphorus, serum intact parathyroid hormone, and serum alkaline phosphatase)

A total of 1226 patients, at 282 dialysis centers, were enrolled between April 1, 2017 and February 28, 2018. Data collection was completed on August 31, 2019. Electronic case report forms were available for 1210 patients, of which 15 were excluded from the safety analysis set and a further three patients were excluded from the efficacy analysis set (Fig. 1). Therefore, the safety analysis set comprised 718 males and 477 females (Table 1). The mean age and dry weight were 64.5 years and 58.7 kg, respectively, and the median dialysis duration was 8.3 years.

**Table 1:** Patient characteristics before the start of etelcalcetide (*N* = 1195)

Characteristic	Value^a^
Male, *n* (%)	718 (60.1)
Female, *n* (%)	477 (39.9)
Age (years)	64.5 ± 12.8
Dry weight (kg)	58.7 ± 15.1 (*n* = 1184)
Dialysis duration (years)	8.3 (4.0–14.4) (*n* = 1168)
Serum iPTH (pg/mL)	346.0 (255.0–481.0) (*n* = 1087)
Serum cCa (mg/dL)	9.23 ± 0.73 (*n* = 1156)
Serum P (mg/mL)	5.84 ± 1.48 (*n* = 1156)
Serum ALP (U/L)	267.0 (208.0–351.0) (*n* = 975)

*iPTH* intact parathyroid hormone, *cCa* corrected calcium, *P* phosphorus, *ALP* alkaline phosphatase

^a^Values are reported as mean ± standard deviation or median (quartile 1–quartile 3)

**Fig. 2:**
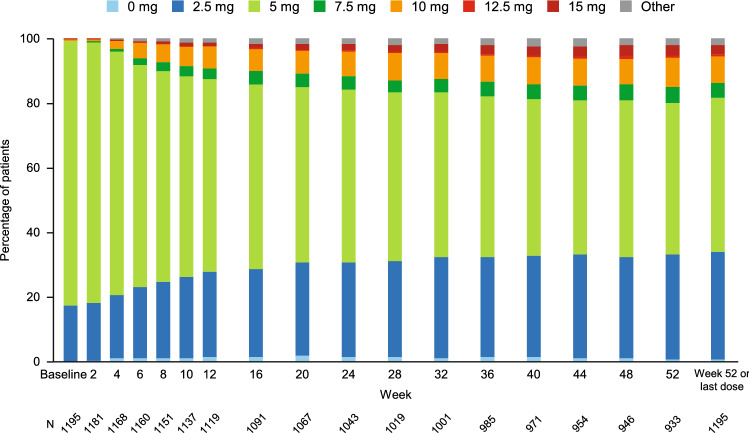
Changes in etelcalcetide doses over time

The starting dose was 5 mg in most of the patients (Fig. 2). The distribution of etelcalcetide doses changed progressively over time, with steady increases in the percentages of patients administered etelcalcetide at a dose of 2.5 mg or ≥ 7.5 mg. The mean dose at Week 52 was 5.1 mg. Overall, 78.1% of patients (933/1195) were treated with etelcalcetide at Week 52. None of the patients received etelcalcetide at a dose of > 0 to < 2.5 mg or at a dose of > 15 mg. At the start of treatment, etelcalcetide was administered three-times-weekly in 1164 patients (97.4%), twice-weekly in 20 patients (1.7%), and once-weekly in 11 patients (0.9%). At Week 52 or the last dosing day, etelcalcetide was administered three-times-weekly in 1078 patients (90.2%), twice-weekly in 64 patients (5.4%), and once-weekly in 47 patients (3.9%), and drug cessation in 6 patients (0.5%). Active vitamin D preparations and anti-hyperphosphatemia drugs were used as concomitant therapies (at least once during the treatment period) in 89.8% and 88.3% of patients, respectively.

### Incidence of ADRs

**Fig. 3:**
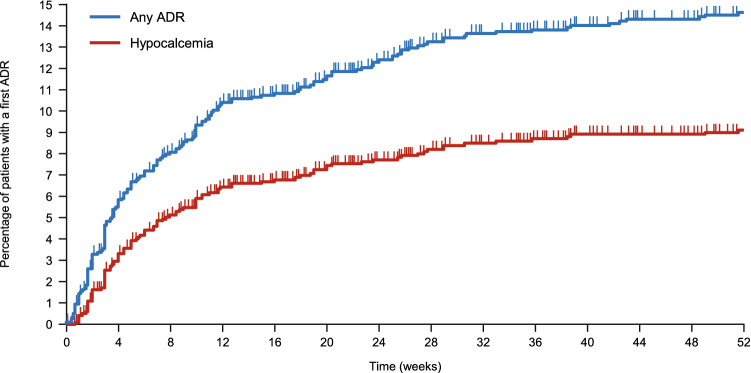
Cumulative incidence of ADR (any) and hypocalcemia (combination of blood calcium decreased or hypocalcemia). The curves were plotted using the Kaplan–Meier method and tick marks indicate censored patients. *ADR* adverse drug reaction

A total of 218 ADRs occurred in 169 patients (14.1%), including 36 serious ADRs in 28 patients (2.3%). Metabolism and nutrition disorders was the most common class of ADRs followed by laboratory tests and gastrointestinal disorders (Table 2). The types of ADRs are summarized in ESM Table 1. ADRs of special interest included ADRs related to hypocalcemia in 104 patients (8.7%), hypersensitivity reactions in six patients (0.5%), QT prolongation in two patients (0.2%), and worsening of cardiac failure and convulsions in one patient each (0.1%) (Table 2). As illustrated in Fig. 3, the cumulative incidence of all ADRs and hypocalcemia increased over the first ~ 32 weeks and then plateaued until the end of the follow-up period.

**Table 2:** ADRs by system organ class and of special interest (*N* = 1195)

Classification	Serious	Non-serious	Total
Number of subjects with ADRs	28	150	169
Number of ADRs	36	182	218
Incidence of ADRs (%)	2.3	12.6	14.1
ADR by system organ class, *n* (%)			
Metabolism and nutrition disorders	1 (0.1)	104 (8.7)	105 (8.8)
Laboratory tests	1 (0.1)	21 (1.8)	22 (1.8)
Gastrointestinal disorders	5 (0.4)	14 (1.2)	19 (1.6)
Nervous system disorders	5 (0.4)	5 (0.4)	10 (0.8)
Musculoskeletal and connective tissue disorders	2 (0.2)	5 (0.4)	7 (0.6)
Skin and subcutaneous tissue disorders	1 (0.1)	6 (0.5)	7 (0.6)
Infections and infestations	3 (0.3)	3 (0.3)	6 (0.5)
Cardiac disorders	3 (0.3)	2 (0.2)	5 (0.4)
General/systemic disorders and administration site conditions	1 (0.1)	3 (0.3)	4 (0.3)
Hepatobiliary disorders	1 (0.1)	2 (0.2)	3 (0.3)
Injury, poisoning and procedural complications	2 (0.2)	1 (0.1)	2 (0.2)
Neoplasms benign, malignant and unspecified	2 (0.2)	–	2 (0.2)
Vascular disorders	2 (0.2)	–	2 (0.2)
Blood and lymphatic system disorders	–	2 (0.2)	2 (0.2)
Ear and labyrinth disorders	1 (0.1)	–	1 (0.1)
Respiratory, thoracic and mediastinal disorders	1 (0.1)	–	1 (0.1)
Eye disorders	–	1 (0.1)	1 (0.1)
Psychiatric disorders	–	1 (0.1)	1 (0.1)
ADRs of special interest, *n* (%)			
ADRs related to hypocalcemia	–	104 (8.7)	104 (8.7)
Hypocalcemia	–	92 (7.7)	92 (7.7)
Blood calcium decreased	–	10 (0.8)	10 (0.8)
Adjusted calcium decreased	–	2 (0.2)	2 (0.2)
Hypersensitivity reactions	1 (0.1)	5 (0.4)	6 (0.5)
Drug eruption	1 (0.1)	1 (0.1)	2 (0.2)
Eczema	–	2 (0.2)	2 (0.2)
Rash	–	1 (0.1)	1 (0.1)
Rash generalized	–	1 (0.1)	1 (0.1)
QT prolongation	1 (0.1)	1 (0.1)	2 (0.2)
Electrocardiogram QT prolonged	1 (0.1)	1 (0.1)	2 (0.2)
Worsening of cardiac failure	1 (0.1)	–	1 (0.1)
Cardiac failure	1 (0.1)	–	1 (0.1)
Convulsions	1 (0.1)	–	1 (0.1)
Epilepsy	1 (0.1)	–	1 (0.1)

*ADR* adverse drug reaction

### Patient- and disease-related factors associated with ADRs and hypocalcemia

Subgroup analyses were performed to identify whether any patient or baseline characteristics were associated with the risk of hypocalcemia or any ADR (Table 3). We found that the incidence of hypocalcemia was significantly higher in patients with a dialysis duration of < 1 or ≥ 1 to < 5 years than in the other subgroups (*P* = 0.002), but there were no differences in the incidence of hypocalcemia among any of the other subgroups assessed. Multiple logistic regression was performed using the following explanatory variables: sex, age, dry weight, baseline serum iPTH, baseline serum cCa, baseline serum P, baseline serum ALP, and dialysis duration. Of these, only age and dialysis duration were independently associated with the risk of hypocalcemia (Table 4). The changes in serum cCa in subgroups of patients who were divided by age or dialysis duration are shown in ESM Figs. 1 and 2. The incidence of any ADRs was significantly associated with dialysis duration (*P* = 0.019), but no differences were found among other subgroups (Table 3).

**Table 3:** Subgroup analysis of hypocalcemia and ADRs (*N* = 1195)

Characteristic			Hypocalcemia			Any ADR		
Variable	Subgroup	*N* (%)	*n*	% (95% CI)	*P*	*n*	% (95% CI)	*P*
All patients		1195 (100.0)	104	8.7 (7.2–10.4)	–	169	14.1 (12.2–16.2)	–
Sex	Male	718 (60.1)	63	8.8 (6.8–11.1)	1.000^a^	101	14.1 (11.6–16.8)	0.933^a^
	Female	477 (39.9)	41	8.6 (6.2–11.5)		68	14.3 (11.2–17.7)	
Age (years)	< 55	281 (23.5)	33	11.7 (8.2–16.1)	0.089^b^	47	16.7 (12.6–21.6)	0.240^b^
	55 to < 65	238 (19.9)	21	8.8 (5.5–13.2)		34	14.3 (10.1–19.4)	
	65 to < 75	422 (35.3)	29	6.9 (4.7–9.7)		53	12.6 (9.6–16.1)	
	≥ 75	254 (21.3)	21	8.3 (5.2–12.4)		35	13.8 (9.8–18.6)	
Dry body weight (kg)	< 40	86 (7.2)	9	10.5 (4.9–18.9)	0.516^b^	15	17.4 (10.1–27.1)	0.159^b^
	40 to < 50	285 (23.8)	24	8.4 (5.5–12.3)		43	15.1 (11.1–19.8)	
	50 to < 60	317 (26.5)	32	10.1 (7.0–14.0)		47	14.8 (11.1–19.2)	
	60 to < 70	255 (21.3)	17	6.7 (3.9–10.5)		31	12.2 (8.4–16.8)	
	70 to < 80	136 (11.4)	13	9.6 (5.2–15.8)		16	11.8 (6.9–18.4)	
	≥ 80	105 (8.8)	8	7.6 (3.3–14.5)		14	13.3 (7.5–21.4)	
	Unknown	11 (0.9)	1	9.1	–	3	27.3	–
Baseline iPTH (pg/mL)	≤ 240	226 (18.9)	10	4.4 (2.1–8.0)	0.104^b^	22	9.7 (6.2–14.4)	0.107^b^
	> 240 to < 500	620 (51.9)	59	9.5 (7.3–12.1)		89	14.4 (11.7–17.4)	
	≥ 500	241 (20.2)	21	8.7 (5.5–13.0)		36	14.9 (10.7–20.1)	
	Unknown	108 (9.0)	14	13.0	–	22	20.4	–
Baseline cCa (mg/dL)	< 8.4	110 (9.2)	10	9.1 (4.4–16.1)	0.523^b^	12	10.9 (5.8–18.3)	0.814^b^
	8.4 to 10.0	904 (75.6)	83	9.2 (7.4–11.3)		136	15.0 (12.8–17.5)	
	> 10.0	142 (11.9)	10	7.0 (3.4–12.6)		18	12.7 (7.7–19.3)	
	Unknown	39 (3.3)	1	2.6	–	3	7.7	–
Baseline P (mg/dL)	< 3.5	34 (2.8)	1	2.9 (0.1–15.3)	0.467^b^	3	8.8 (1.9–23.7)	0.859^b^
	3.5 to 6.0	655 (54.8)	58	8.9 (6.8–11.3)		96	14.7 (12.0–17.6)	
	> 6.0	467 (39.1)	44	9.4 (6.9–12.4)		67	14.3 (11.3–17.9)	
	Unknown	39 (3.3)	1	2.6	–	3	7.7	–
Baseline ALP (U/L)	< 160	87 (7.3)	12	13.8 (7.3–22.9)	0.965^b^	17	19.5 (11.8–29.4)	0.593^b^
	≥ 160 to < 320	566 (47.4)	50	8.8 (6.6–11.5)		76	13.4 (10.7–16.5)	
	≥ 320	322 (26.9)	34	10.6 (7.4–14.4)		55	17.1 (13.1–21.6)	
	Unknown	220 (18.4)	8	3.6	–	21	9.5	–
Dialysis duration (years)	< 1	85 (7.1)	10	11.8 (5.8–20.6)	0.002^b^	17	20.0 (12.1–30.1)	0.019^b^
	≥ 1 to < 5	278 (23.3)	39	14.0 (10.2–18.7)		44	15.8 (11.7–20.7)	
	≥ 5 to < 10	314 (26.3)	21	6.7 (4.2–10.0)		46	14.6 (10.9–19.1)	
	≥ 10 to < 20	362 (30.3)	26	7.2 (4.7–10.3)		48	13.3 (9.9–17.2)	
	≥ 20	129 (10.8)	7	5.4 (2.2–10.9)		11	8.5 (4.3–14.7)	
	Unknown	27 (2.3)	1	3.7	–	3	11.1	–
Dialysis modality	HD	795 (66.5)	67	8.4 (6.6–10.6)	0.664^a^	108	13.6 (11.3–16.2)	0.430^a^
	HDF	400 (33.5)	37	9.3 (6.6–12.5)		61	15.3 (11.9–19.2)	
Dialysate Ca (mEq/L)	< 2.5	7 (0.6)	0	0.0 (0.0–41.0)	0.449^b^	0	0.0 (0.0–41.0)	0.507^b^
	2.5	386 (32.3)	30	7.8 (5.3–10.9)		50	13.0 (9.8–16.7)	
	> 2.5 to < 2.75	8 (0.7)	0	0.0 (0.0–36.9)		0	0.0 (0.0–36.9)	
	2.75	414 (34.6)	38	9.2 (6.6–12.4)		64	15.5 (12.1–19.3)	
	3.0	355 (29.7)	32	9.0 (6.2–12.5)		50	14.1 (10.6–18.1)	
	> 3.0	1 (0.1)	0	0.0 (0.0–97.5)		0	0.0 (0.0–97.5)	
	Unknown	24 (2.0)	4	16.7	–	5	20.8	–
Time since last dose of cinacalcet (days)	< 7	396 (33.1)	29	7.3 (5.0–10.3)	0.146^b^	52	13.1 (10.0–16.9)	0.704^b^
	≥ 7 to < 28	137 (11.5)	11	8.0 (4.1–13.9)		24	17.5 (11.6–24.9)	
	≥ 28/cinacalcet-naïve	647 (54.1)	64	9.9 (7.7–12.5)		93	14.4 (11.8–17.3)	
	Unknown	15 (1.3)	0	0.0	–	0	0.0	–
Last daily dose of cinacalcet (mg)^c^	≤ 25	355 (56.7)	28	7.9 (5.3–11.2)	0.689^b^	49	13.8 (10.4–17.8)	0.992^b^
	> 25 to ≤ 50	135 (21.6)	12	8.9 (4.7–15.0)		26	19.3 (13.0–26.9)	
	> 50 to ≤ 75	80 (12.8)	5	6.3 (2.1–14.0)		10	12.5 (6.2–21.8)	
	> 75 to ≤ 100	51 (8.1)	3	5.9 (1.2–16.2)		5	9.8 (3.3–21.4)	
	Unknown	5 (0.8)	0	0.0	–	0	0.0	–
Liver disease	No	1110 (92.9)	94	8.5 (6.9–10.3)	0.063^a^	154	13.9 (11.9–16.0)	0.093^a^
	Yes	63 (5.3)	10	15.9 (7.9–27.3)		14	22.2 (12.7–34.5)	
	Unknown	22 (1.8)	0	0.0	–	1	4.5	–
Diabetes mellitus	No	757 (63.3)	69	9.1 (7.2–11.4)	0.525^a^	113	14.9 (12.5–17.7)	0.343^a^
	Yes	438 (36.7)	35	8.0 (5.6–10.9)		56	12.8 (9.8–16.3)	

*ADR* adverse drug reaction, *CI* confidence interval, *iPTH* intact parathyroid hormone, *cCa* corrected calcium, *P* phosphorus, *ALP* alkaline phosphatase, *HD* hemodialysis, *HDF* hemodiafiltration

^a^Fisher’s exact test

^b^Wilcoxon rank sum test

^c^*N* = 626

**Table 4:** Multiple logistic regression analyses of factors associated with the occurrence of hypocalcemia in the safety analysis set or achieving iPTH within the target range (60–240 pg/mL) in the efficacy analysis set

Outcome: Hypocalcemia	*N*	Cases (%)	OR (95% CI)	*P*
Total	899	83 (9.2)		
Age				
< 65 years	397	47 (11.8)	Reference	
≥ 65 years	502	36 (7.2)	0.578 (0.365–0.915)	0.019
Dialysis duration				
< 5 years	269	40 (14.9)	Reference	
≥ 5 years	630	43 (6.8)	0.421 (0.266–0.666)	< 0.001

Outcome: iPTH within the target range	*N*	Cases (%)	OR (95% CI)	*P*
Total	599	390 (65.1)		
iPTH at baseline				
≤ 240 pg/mL	108	78 (72.2)	Reference	
> 240 to < 500 pg/mL	359	243 (67.7)	0.806 (0.501–1.296)	0.373
≥ 500 pg/mL	132	69 (52.3)	0.421 (0.245–0.724)	0.002

For both outcomes, logistic regression was performed using the forward–backward stepwise selection method with the following explanatory variables: sex, age, dry weight, baseline serum iPTH, baseline cCa, baseline P, baseline ALP, and dialysis duration.

*OR* odds ratio, *CI* confidence interval, *iPTH* intact parathyroid hormone, *cCa* corrected calcium, *P* phosphorus, *ALP* alkaline phosphatase

### Efficacy of etelcalcetide

**Fig. 4:**
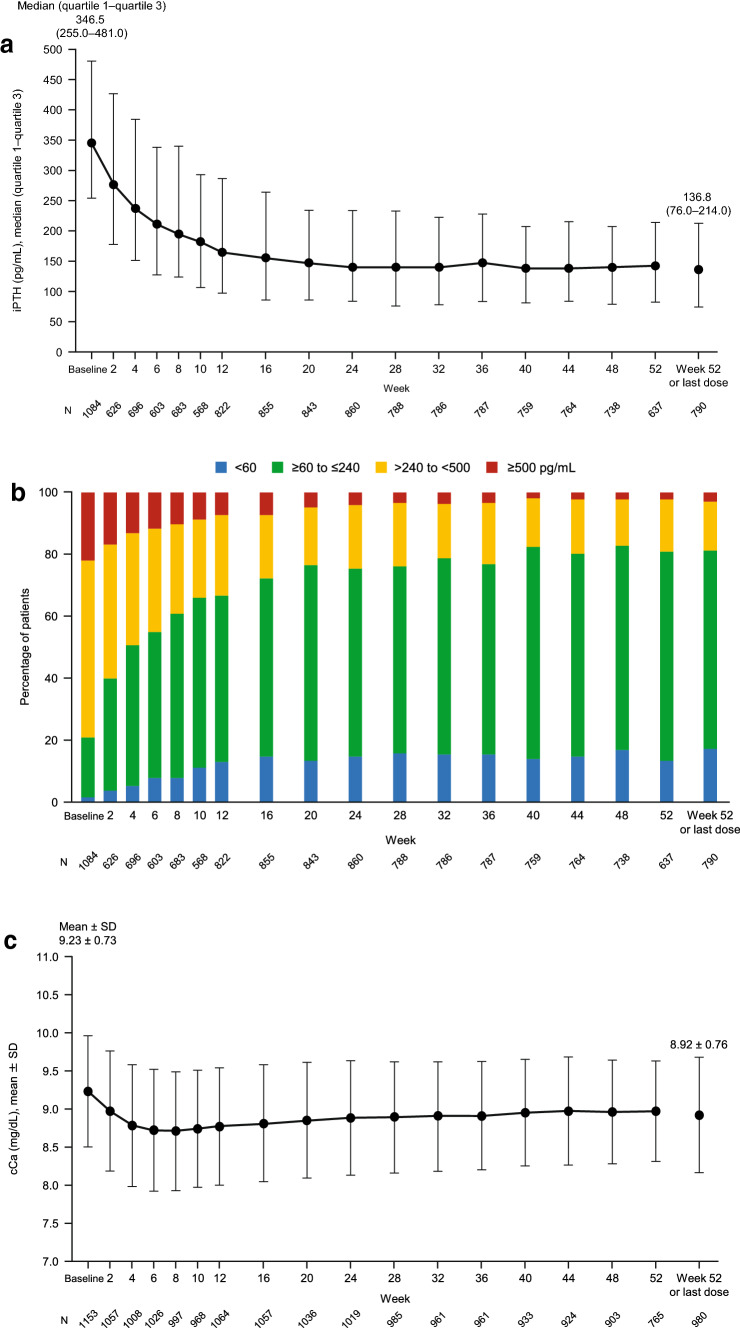

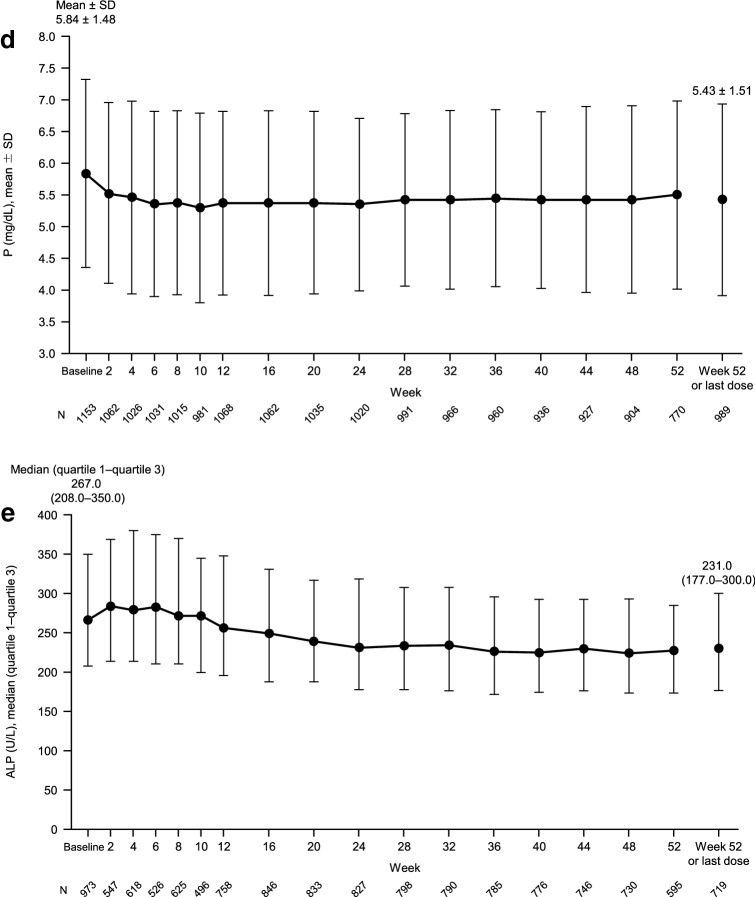
Changes in serum iPTH levels (**a**), distribution of serum iPTH levels (**b**), serum cCa (**c**), serum P (**d**), and serum ALP (**e**) over time. Values are median (quartile 1–quartile 3; **a**, **e**), percentage of patients (**b**), or mean ± standard deviation (**c**, **d**). *iPTH* intact parathyroid hormone, *SD* standard deviation, *cCa* corrected calcium, *P* phosphorus, *ALP* alkaline phosphatase

The efficacy of etelcalcetide was examined in terms of the changes in serum iPTH, cCa, P, and ALP levels over time. As shown in Fig. 4a, the serum iPTH level decreased steadily during the follow-up period, reaching a median (quartile 1–quartile 3) of 136.8 (76.0–214.0) pg/mL at Week 52 or last dose. In accordance with these changes in the median serum iPTH level, the proportion of patients who achieved serum iPTH within the target range of 60–240 pg/mL steadily increased from 19.5% at Week 0 to 64.1% at Week 52 or last dose (Fig. 4b). Although the proportion of responders was significantly greater among patients with a baseline serum iPTH level ≤ 240 pg/mL (72.8%) than in patients with higher baseline levels (*P* < 0.001), the proportion was over 50% among patients with baseline levels of > 240 to < 500 pg/mL (67.8%) or ≥ 500 pg/mL (53.5%) (Table 5). The proportion of responders was also significantly greater in patients with low baseline serum ALP levels (*P* = 0.044) (Table 5).

**Table 5:** Subgroup analysis of patients achieving iPTH within the target range (60–240 pg/mL) at Week 52 or last dose (*N* = 790)

Characteristic			Proportion achieving target range of iPTH		
Variable	Subgroup	*N* (%)	*n*	% (95% CI)	*P*
All patients		790 (100.0)	506	64.1 (60.6–67.4)	–
Sex	Male	459 (58.1)	292	63.6 (59.0–68.0)	0.822^a^
	Female	331 (41.9)	214	64.7 (59.2–69.8)	
Age (years)	< 55	189 (23.9)	130	68.8 (61.7–75.3)	0.621^b^
	55 to < 65	159 (20.1)	93	58.5 (50.4–66.2)	
	65 to < 75	282 (35.7)	180	63.8 (57.9–69.4)	
	≥ 75	160 (20.3)	103	64.4 (56.4–71.8)	
Dry body weight (kg)	< 40	57 (7.2)	37	64.9 (51.1–77.1)	0.364^b^
	40 to < 50	193 (24.4)	127	65.8 (58.6–72.5)	
	50 to < 60	205 (25.9)	134	65.4 (58.4–71.9)	
	60 to < 70	173 (21.9)	109	63.0 (55.3–70.2)	
	70 to < 80	87 (11.0)	52	59.8 (48.7–70.1)	
	≥ 80	70 (8.9)	44	62.9 (50.5–74.1)	
	Unknown	5 (0.6)	3	60.0	–
Baseline iPTH (pg/mL)	≤ 240	147 (18.6)	107	72.8 (64.8–79.8)	< 0.001^b^
	> 240 to < 500	428 (54.2)	290	67.8 (63.1–72.2)	
	≥ 500	159 (20.1)	85	53.5 (45.4–61.4)	
	Unknown	56 (7.1)	24	42.9	–
Baseline cCa (mg/dL)	< 8.4	67 (8.5)	43	64.2 (51.5–75.5)	0.278^b^
	8.4 to 10.0	606 (76.7)	388	64.0 (60.1–67.9)	
	> 10.0	88 (11.1)	63	71.6 (61.0–80.7)	
	Unknown	29 (3.7)	12	41.4	–
Baseline P (mg/dL)	< 3.5	20 (2.5)	11	55.0 (31.5–76.9)	0.429^b^
	3.5 to 6.0	427 (54.1)	275	64.4 (59.7–68.9)	
	> 6.0	314 (39.7)	208	66.2 (60.7–71.5)	
	Unknown	29 (3.7)	12	41.4	–
Baseline ALP (U/L)	< 160	56 (7.1)	39	69.6 (55.9–81.2)	0.044^b^
	≥ 160 to < 320	356 (45.1)	240	67.4 (62.3–72.3)	
	≥ 320	215 (27.2)	128	59.5 (52.6–66.2)	
	Unknown	163 (20.6)	99	60.7	–
Dialysis duration (years)	< 1	60 (7.6)	32	53.3 (40.0–66.3)	0.518^b^
	≥ 1 to < 5	177 (22.4)	117	66.1 (58.6–73.0)	
	≥ 5 to < 10	208 (26.3)	136	65.4 (58.5–71.8)	
	≥ 10 to < 20	248 (31.4)	156	62.9 (56.6–68.9)	
	≥ 20	81 (10.3)	55	67.9 (56.6–77.8)	
	Unknown	16 (2.0)	10	62.5	–
Dialysis modality	HD	538 (68.1)	349	64.9 (60.7–68.9)	0.525^a^
	HDF	252 (31.9)	157	62.3 (56.0–68.3)	
Dialysate Ca (mEq/L)	< 2.5	2 (0.3)	1	50.0 (1.3–98.7)	0.931^b^
	2.5	250 (31.6)	162	64.8 (58.5–70.7)	
	> 2.5 to < 2.75	4 (0.5)	3	75.0 (19.4–99.4)	
	2.75	272 (34.4)	168	61.8 (55.7–67.6)	
	3.0	244 (30.9)	159	65.2 (58.8–71.1)	
	> 3.0	0 (0.0)	0	–	
	Unknown	18 (2.3)	13	72.2	–
Time since last dose of cinacalcet (days)	< 7	263 (33.3)	176	66.9 (60.9–72.6)	0.141^b^
	≥ 7 to < 28	89 (11.3)	61	68.5 (57.8–78.0)	
	≥ 28/cinacalcet-naïve	427 (54.1)	264	61.8 (57.0–66.5)	
	Unknown	11 (1.4)	5	45.5	–
Last daily dose of cinacalcet (mg)^c^	≤ 25	237 (57.2)	161	67.9 (61.6–73.8)	0.944^b^
	> 25 to ≤ 50	86 (20.8)	54	62.8 (51.7–73.0)	
	> 50 to ≤ 75	53 (12.8)	41	77.4 (63.8–87.7)	
	> 75 to ≤ 100	36 (8.7)	22	61.1 (43.5–76.9)	
	Unknown	2 (0.5)	1	50.0	–
Liver disease	No	733 (92.8)	470	64.1 (60.5–67.6)	0.423^a^
	Yes	44 (5.6)	31	70.5 (54.8–83.2)	
	Unknown	13 (1.6)	5	38.5	–
Diabetes mellitus	No	512 (61.8)	340	66.4 (62.1–70.5)	0.063^a^
	Yes	278 (35.2)	166	59.7 (53.7–65.5)	

*iPTH* intact parathyroid hormone, *CI* confidence interval, *cCa* corrected calcium, *P* phosphorus, *ALP* alkaline phosphatase, *HD* hemodialysis, *HDF* hemodiafiltration

^a^Fisher’s exact test

^b^Wilcoxon rank sum test

^c^*N* = 414

Multiple logistic regression was performed to identify possible factors associated with achievement of serum iPTH within the target range using the following explanatory variables: sex, age, dry weight, baseline iPTH, baseline cCa, baseline P, baseline ALP, and dialysis duration. Of these, only the baseline iPTH level was independently associated with the proportion of patients who achieved iPTH within the target range (Table 4).

In terms of other efficacy outcomes, we found that the mean serum cCa and P levels remained within their target ranges throughout the follow-up period (Fig. 4c, d). We also observed improvements in serum ALP levels (Fig. 4e).

This PMS enrolled patients who were ineligible for the previous long-term clinical study in Japan (52-week study) [7], including patients whose last dose of cinacalcet was < 28 days before the start of etelcalcetide, patients with a baseline serum iPTH of ≤ 240 pg/mL, and patients with a baseline serum cCa of < 8.4 mg/dL. The changes in serum iPTH, cCa, P, and ALP in these subgroups of patients are shown in ESM Figs. 3–5.

## Discussion

This was the first real-world, large-scale, long-term observational PMS to examine the safety and efficacy of etelcalcetide for the management of SHPT. The rates of ADRs (14.1%) and serious ADRs (2.3%) do not exceed the levels reported in the 52-week study (ADRs, 27.9%; serious ADRs, 2.1%) [7].

Hypocalcemia, a calcimimetic-related AE, was the most common ADR as hypocalcemia-related ADRs occurred in 104 patients (8.7%). Other ADRs of special interest included hypersensitivity reactions, worsening of cardiac failure, QT prolongation, and convulsions, all of which were infrequent and have already been described in the package insert for etelcalcetide. It was suggested in earlier studies that the injectable formulation of etelcalcetide might contribute to a lower rate or decreased severity [7, 8] of gastrointestinal disorder-related ADRs, and a similar trend was observed in the present PMS.

From an efficacy perspective, we observed gradual reductions in serum iPTH that were not too rapid. At Week 52 or last dose, 64.1% of patients had a serum iPTH of 60–240 pg/mL. In the prior 52-week study, 87.5% of patients achieved serum iPTH levels of 60–240 pg/mL [7]. The higher proportion of patients achieving target iPTH levels in that study is likely due to the treatment protocol, which required adjustment of the etelcalcetide dose according to the patient’s iPTH level to reach the target range. These improvements in serum iPTH levels were also associated with maintenance of serum cCa and P levels within appropriate ranges, and an improvement in the serum ALP level. Taken together, these findings demonstrate that long-term administration of etelcalcetide improves the metabolic state of patients with SHPT [7, 9] and the improvement in ALP, in particular, is expected to lower the risk of bone disorders and vascular calcification.

We also assessed which baseline factors were associated with achieving target iPTH levels by performing multiple logistic regression. In this analysis, the baseline iPTH level was the only explanatory factor. We found that patients with higher serum iPTH levels at baseline were less likely to achieve iPTH levels within the target range. Furthermore, it took longer for serum iPTH levels to decrease towards the target range in patients with higher baseline iPTH (ESM Fig. 4).

The 52-week study [7] excluded patients whose last dose of cinacalcet was < 28 days before starting etelcalcetide. In these patients, the serum iPTH levels were lower at baseline and decreased less efficiently than in patients whose last dose of cinacalcet was ≥ 28 days earlier and in treatment-naïve patients (ESM Fig. 3). These trends may be due to a carryover effect of prior cinacalcet.

Parathyroid gland hyperplasia has emerged as a determinant of unsuccessful treatment of SHPT, including poor outcomes of calcimimetics [10, 11]. A recent study revealed that dialysis duration, parathyroid gland volume, and serum PTH were risk factors for nodular hyperplasia of parathyroid glands in SHPT [12]. In this PMS, we found that patients with a shorter history of dialysis were more likely to experience hypocalcemia and tended to have lower serum cCa levels, particularly in the first 12 weeks (ESM Fig. 1), but the efficacy of etelcalcetide was comparable (about 60–70%) in subgroups regardless of dialysis duration. The higher rate of hypocalcemia might be due to higher sensitivity of the parathyroid glands, resulting in greater propensity for lowering Ca levels. Further studies might be necessary to evaluate the impact of parathyroid gland hyperplasia on the clinical efficacy of etelcalcetide in patients with a long dialysis duration. The serum cCa levels also tracked slightly lower in younger patients (ESM Fig. 2), which might reflect a relationship between younger age and greater parathyroid gland sensitivity/PTH secretory potential [13–16], although this has yet to be confirmed in the context of SHPT.

As in the prior 52-week study [17], we found no differences in the efficacy and safety of etelcalcetide when patients were divided into subgroups according to their dialysate Ca concentration (Tables 3 and 5).

The results of this PMS support those of the 52-week study, highlighting the long-term tolerability and efficacy of etelcalcetide in real-life clinical practice. Nevertheless, there are some slight differences that might be related to differences in patient eligibility. In particular, this PMS enrolled patients in real-life clinical practice and included patients who were ineligible for the prior study (e.g., last dose of cinacalcet < 28 days before starting etelcalcetide and baseline serum cCa < 8.4 mg/dL), and there were no specific criteria for adjusting the dose of etelcalcetide or concomitantly used drugs unlike the 52-week study [7].

The real-life use of etelcalcetide was also evaluated in a European observational study performed at 23 dialysis centers in Italy, in which clinicians took into account the KDIGO guidelines to maintain Ca, P, and PTH levels [8]. Etelcalcetide was administered according to its package insert and started at a dose of 5 mg three-times-weekly, or at another dose if deemed appropriate by the attending clinician. The doses of concomitant medications could also be adjusted if necessary. Of 1190 patients enrolled, 168 received etelcalcetide (5 mg three-times-weekly), which included 56 calcimimetic-naïve patients. The authors reported low rates of hypocalcemia and gastrointestinal disorders. The serum iPTH level decreased from a median of 636 to 357 pg/mL, while the proportion of responders (defined as iPTH of 150–300 pg/mL) increased from 27 to 63%. Significant reductions in serum Ca, P, and ALP levels were also observed in that study. Since the patient background and management of SHPT differ between European and Japanese studies (e.g., baseline iPTH, target range), we cannot directly compare the results of these studies. Nevertheless, their results of an observational study in a real-world setting provide further evidence in support of our findings, indicating that etelcalcetide is a tolerable and effective intravenous drug for the management of iPTH levels in patients with SHPT on hemodialysis.

### Limitations

The main limitations of this surveillance include its non-randomized single arm design and that efficacy data were not available for all patients. However, these limitations are partly offset by the large sample size and long-term observation, which enable the detection of rare ADRs and our ability to monitor efficacy over a longer period of time than was done in many earlier studies.

## Conclusion

In conclusion, this was the first real-world, large-scale, long-term observational surveillance of etelcalcetide in Japan and involved a much larger cohort of patients than enrolled in prior clinical trials. We did not observe any new safety concerns. Etelcalcetide was associated with clinically relevant improvements in serum iPTH levels together with maintenance of serum cCa, P, and ALP levels. Intravenous administration of etelcalcetide is a clinically useful treatment for SHPT that avoids the pill burden associated with other treatments and its compliance can be maintained via administration at the dialysis center.

## Electronic supplementary material

Below is the link to the electronic supplementary material.

Supplementary file1 (PDF 519 kb)

## Data Availability

Qualified researchers may request Ono Pharma (ONO) to disclose individual patient-level data from clinical studies through the following website: https://www.clinicalstudydatarequest.com. For more information on ONO’s Policy for the Disclosure of Clinical Study Data, please see the following website: https://www.ono.co.jp/eng/rd/policy.html.
