# Supplementary material for: Safety and efficacy of etelcalcetide, an intravenous calcimimetic, for up to 52 weeks in hemodialysis patients with secondary hyperparathyroidism: results of a post-marketing surveillance in Japan
Source: Clin Exp Nephrol. 2020 Aug 20;25(1):66–79. doi: 10.1007/s10157-020-01936-2 (PMC7794109; doi:10.1007/s10157-020-01936-2)
Supplement: Supplementary file 1 — Supplementary file1 (PDF 519 kb) [file 10157_2020_1936_MOESM1_ESM.pdf]

Journal: Clinical and Experimental Nephrology

ELECTRONIC SUPPLEMENTARY MATERIAL

Safety and efficacy of etelcalcetide, an intravenous calcimimetic, for up to 52 weeks in hemodialysis patients with secondary hyperparathyroidism: Results of a post-marketing surveillance in Japan

Keitaro Yokoyama, Masafumi Fukagawa, Takashi Shigematsu, Takashi Akiba, Ken Yoshikawa, Akira Tsuchiya, Misato Kuwabara, Tadao Akizawa

Corresponding author

Keitaro Yokoyama

Harumi Triton Clinic, The Jikei University Hospital, Tokyo, Japan,

E-mail: keitaro@jikei.ac.jp

Contents

ESM Table 1 Adverse drug reactions by system organ class/preferred term

ESM Fig. 1 Changes in serum cCa according to dialysis duration

ESM Fig. 2 Changes in serum cCa according to age at baseline

ESM Fig. 3 Changes in serum iPTH (a), cCa (b), P (c) and ALP (d) levels according to the time between the last dose of cinacalcet and the start of etelcalcetide

ESM Fig. 4 Changes in serum iPTH (a), cCa (b), P (c) and ALP (d) according to baseline iPTH level

ESM Fig. 5 Changes in serum iPTH (a), cCa (b), P (c) and ALP (d) levels according to baseline Ca level

ESM Table 1 Adverse drug reactions by system organ class/preferred term (N = 1195)

Classification	Serious	Non-serious	Total
**Number of subjects with ADRs**	**28**	**150**	**169**
**Number of ADRs**	**36**	**182**	**218**
**Incidence of ADRs (%)**	**2.3**	**12.6**	**14.1**
**ADR by system organ class and preferred term, n (%)**			
**Metabolism and nutrition disorders**	**1 (0.1)**	**104 (8.7)**	**105 (8.8)**
Hypocalcemia	–	92 (7.7)	92 (7.7)
Hyperphosphatemia	–	6 (0.5)	6 (0.5)
Hypophosphatemia	–	4 (0.3)	4 (0.3)
Decreased appetite	–	3 (0.3)	3 (0.3)
Hypercalcemia	–	3 (0.3)	3 (0.3)
Hypophagia	1 (0.1)	–	1 (0.1)
Hyperglycemia	–	1 (0.1)	1 (0.1)
Tetany	–	1 (0.1)	1 (0.1)
**Laboratory tests**	**1 (0.1)**	**21 (1.8)**	**22 (1.8)**
Blood calcium decreased	–	10 (0.8)	10 (0.8)
Electrocardiogram QT prolonged	1 (0.1)	1 (0.1)	2 (0.2)
Adjusted calcium decreased	–	2 (0.2)	2 (0.2)
Blood parathyroid hormone decreased	–	2 (0.2)	2 (0.2)
Blood parathyroid hormone increased	–	2 (0.2)	2 (0.2)
Blood phosphorus decreased	–	1 (0.1)	1 (0.1)
Blood phosphorus increased	–	1 (0.1)	1 (0.1)
Blood pressure increased	–	1 (0.1)	1 (0.1)
Weight increased	–	1 (0.1)	1 (0.1)
**Gastrointestinal disorders**	**5 (0.4)**	**14 (1.2)**	**19 (1.6)**
Nausea	1 (0.1)	10 (0.8)	11 (0.9)
Vomiting	1 (0.1)	3 (0.3)	4 (0.3)
Abdominal discomfort	–	3 (0.3)	3 (0.3)

Gastrointestinal hemorrhage	2 (0.2)	–	2 (0.2)
Enterocolitis	1 (0.1)	–	1 (0.1)
Gastritis erosive	1 (0.1)	–	1 (0.1)
Pancreatitis acute	1 (0.1)	–	1 (0.1)
Large intestine polyp	1 (0.1)	–	1 (0.1)
Diarrhea	–	1 (0.1)	1 (0.1)
Gastritis	–	1 (0.1)	1 (0.1)
**Nervous system disorders**	**5 (0.4)**	**5 (0.4)**	**10 (0.8)**
Dizziness	–	3 (0.3)	3 (0.3)
Cerebral bleeding	1 (0.1)	–	1 (0.1)
Epilepsy	1 (0.1)	–	1 (0.1)
Facial paralysis	1 (0.1)	–	1 (0.1)
Putamen hemorrhage	1 (0.1)	–	1 (0.1)
Thrombotic cerebral infarction	1 (0.1)	–	1 (0.1)
Hypoesthesia	–	1 (0.1)	1 (0.1)
Taste disorder	–	1 (0.1)	1 (0.1)
**Musculoskeletal and connective tissue disorders**	**2 (0.2)**	**5 (0.4)**	**7 (0.6)**
Muscle spasms	–	2 (0.2)	2 (0.2)
Systemic lupus erythematosus	1 (0.1)	–	1 (0.1)
Spinal column stenosis	1 (0.1)	–	1 (0.1)
Bursitis	–	1 (0.1)	1 (0.1)
Osteoarthritis	–	1 (0.1)	1 (0.1)
Periarthritis	–	1 (0.1)	1 (0.1)
**Skin and subcutaneous tissue disorders**	**1 (0.1)**	**6 (0.5)**	**7 (0.6)**
Drug eruption	1 (0.1)	1 (0.1)	2 (0.2)
Eczema	–	2 (0.2)	2 (0.2)
Pruritus	–	2 (0.2)	2 (0.2)
Rash	–	1 (0.1)	1 (0.1)
Rash generalized	–	1 (0.1)	1 (0.1)
**Infections and infestations**	**3 (0.3)**	**3 (0.3)**	**6 (0.5)**

Infectious pleural effusion	1 (0.1)	–	1 (0.1)
Pneumonia	1 (0.1)	–	1 (0.1)
Septic shock	1 (0.1)	–	1 (0.1)
Shunt infection	1 (0.1)	–	1 (0.1)
Folliculitis	–	1 (0.1)	1 (0.1)
Dermatophytosis of nail	–	1 (0.1)	1 (0.1)
Rhinopharyngitis	–	1 (0.1)	1 (0.1)
**Cardiac disorders**	**3 (0.3)**	**2 (0.2)**	**5 (0.4)**
Atrial fibrillation	1 (0.1)	1 (0.1)	2 (0.2)
Angina pectoris	1 (0.1)	–	1 (0.1)
Cardiac failure	1 (0.1)	–	1 (0.1)
Myocardial ischemia	1 (0.1)	–	1 (0.1)
Supraventricular tachycardia	–	1 (0.1)	1 (0.1)
**General/systemic disorders and administration site conditions**	**1 (0.1)**	**3 (0.3)**	**4 (0.3)**
Death	1 (0.1)	–	1 (0.1)
Chest discomfort	–	1 (0.1)	1 (0.1)
Chills	–	1 (0.1)	1 (0.1)
Feeling hot	–	1 (0.1)	1 (0.1)
Pyrexia	–	1 (0.1)	1 (0.1)
**Hepatobiliary disorders**	**1 (0.1)**	**2 (0.2)**	**3 (0.3)**
Cholecystitis acute	1 (0.1)	–	1 (0.1)
Hepatic function abnormal	–	1 (0.1)	1 (0.1)
Liver disorder	–	1 (0.1)	1 (0.1)
**Injury, poisoning and procedural complications**	**2 (0.2)**	**1 (0.1)**	**2 (0.2)**
Shunt occlusion	1 (0.1)	–	1 (0.1)
Spinal compression fracture	1 (0.1)	–	1 (0.1)
Shunt stenosis	–	1 (0.1)	1 (0.1)
**Neoplasms benign, malignant and unspecified**	**2 (0.2)**	**–**	**2 (0.2)**
Angiosarcoma	1 (0.1)	–	1 (0.1)

Bladder cancer	1 (0.1)	–	1 (0.1)
**Vascular disorders**	**2 (0.2)**	–	**2 (0.2)**
Peripheral arterial occlusive disease	1 (0.1)	–	1 (0.1)
Peripheral ischemia	1 (0.1)	–	1 (0.1)
**Blood and lymphatic system disorders**	**–**	**2 (0.2)**	**2 (0.2)**
Anemia	–	1 (0.1)	1 (0.1)
Nephrogenic anemia	–	1 (0.1)	1 (0.1)
**Ear and labyrinth disorders**	**1 (0.1)**	**–**	**1 (0.1)**
Sudden deafness	1 (0.1)	–	1 (0.1)
**Respiratory, thoracic and mediastinal disorders**	**1 (0.1)**	**–**	**1 (0.1)**
Hypoxia	1 (0.1)	–	1 (0.1)
**Eye disorders**	**–**	**1 (0.1)**	**1 (0.1)**
Corneal erosion	–	1 (0.1)	1 (0.1)
**Psychiatric disorders**	**–**	**1 (0.1)**	**1 (0.1)**
Dysphoria	–	1 (0.1)	1 (0.1)

ADR adverse drug reaction

ESM Figure legends

ESM Fig. 1 Changes in serum cCa according to dialysis duration. *cCa* corrected calcium

ESM Fig. 2 Changes in serum cCa according to age at baseline. *cCa* corrected calcium

ESM Fig. 3 Changes in serum iPTH (a), cCa (b), P (c) and ALP (d) levels according to the time between the last dose of cinacalcet and the start of etelcalcetide. *iPTH* intact parathyroid hormone, *cCa* corrected calcium, *P* phosphorus, *ALP* alkaline phosphatase

ESM Fig. 4 Changes in serum iPTH (a), cCa (b), P (c) and ALP (d) according to baseline iPTH level. *iPTH* intact parathyroid hormone, *cCa* corrected calcium, *P* phosphorus, *ALP* alkaline phosphatase

ESM Fig. 5 Changes in serum iPTH (a), cCa (b), P (c) and ALP (d) levels according to baseline cCa level. *iPTH* intact parathyroid hormone, *cCa* corrected calcium, *P* phosphorus, *ALP* alkaline phosphatase

ESM Fig. 1 Changes in serum cCa according to dialysis duration

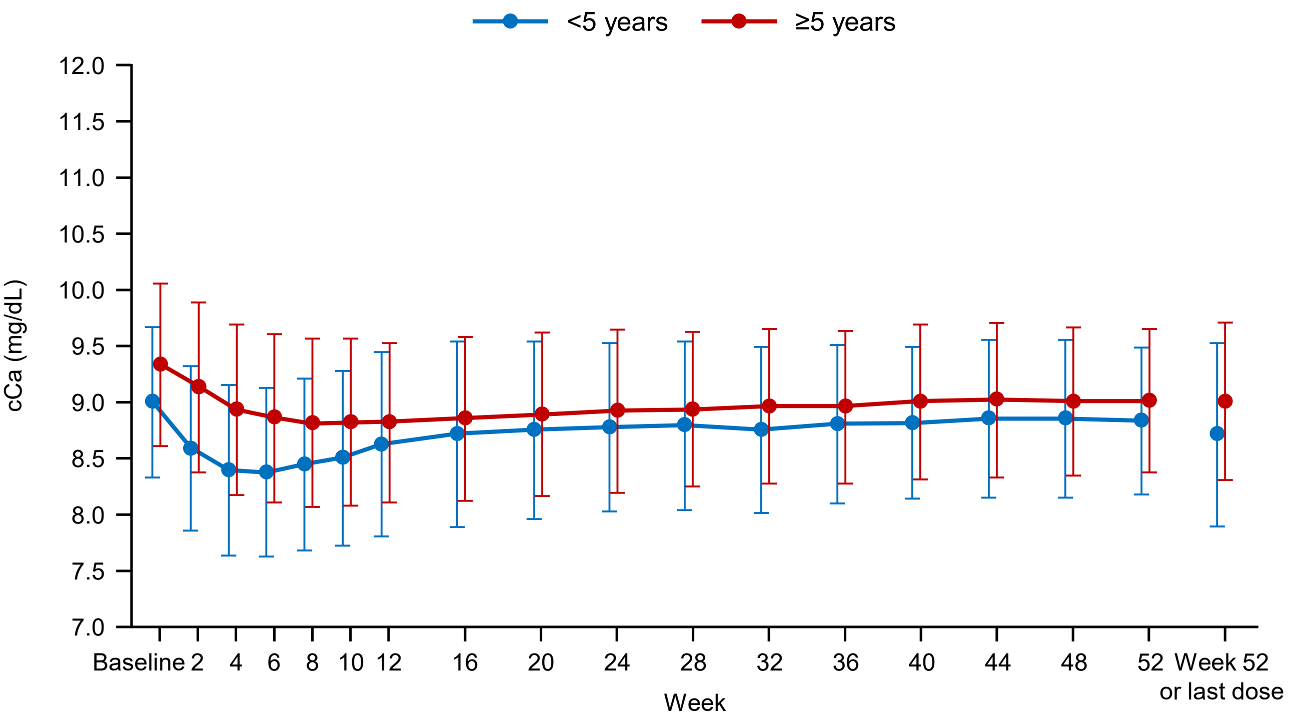

N																	
<5 years	347	324	300	310	296	289	317	309	301	289	284	270	279	262	261	256	214	293
≥5 years	782	708	687	692	675	658	722	725	712	710	681	670	661	651	642	626	538	668

ESM Fig. 2 Changes in serum cCa according to age at baseline

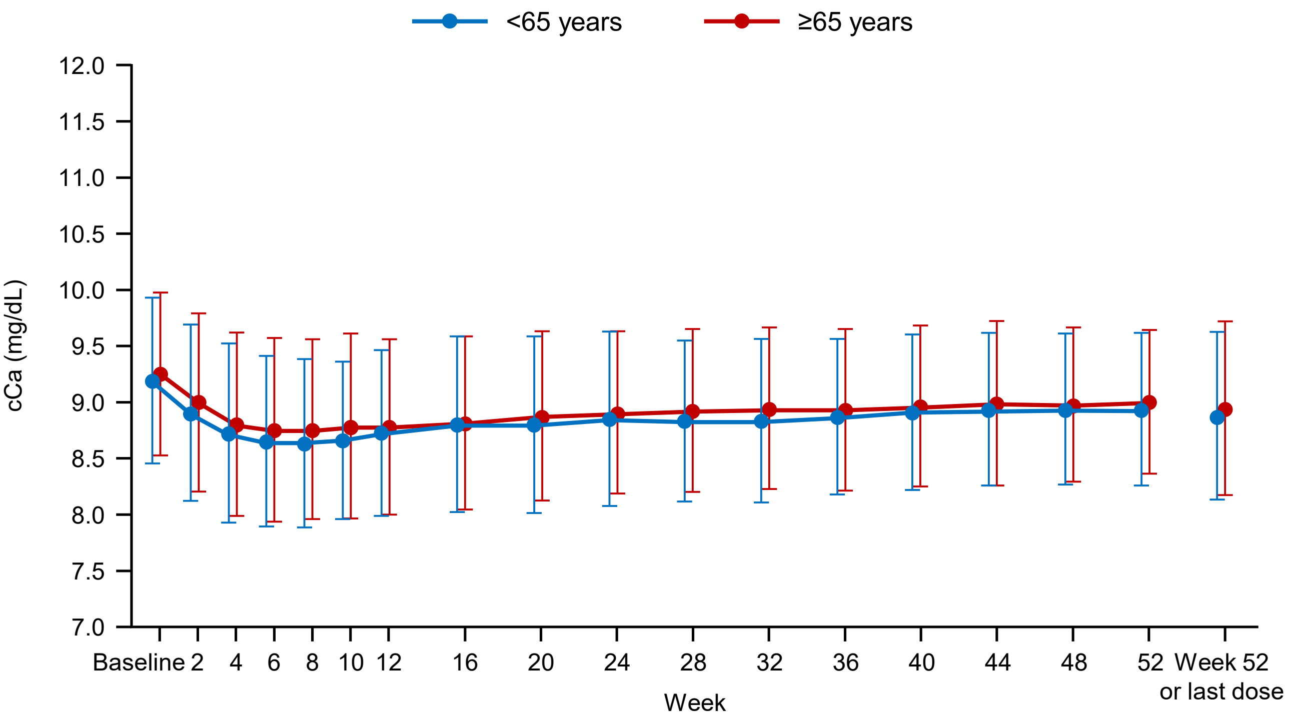

N																	
<65 years	499	456	444	447	434	419	469	468	458	451	443	430	435	426	425	422	356	426
≥65 years	657	601	564	579	563	549	595	589	578	568	542	531	526	507	499	481	409	554

ESM Fig. 3 Changes in serum iPTH (a), cCa (b), P (c) and ALP (d) levels according to the time between the last dose of cinacalcet and the start of etelcalcetide

a. iPTH

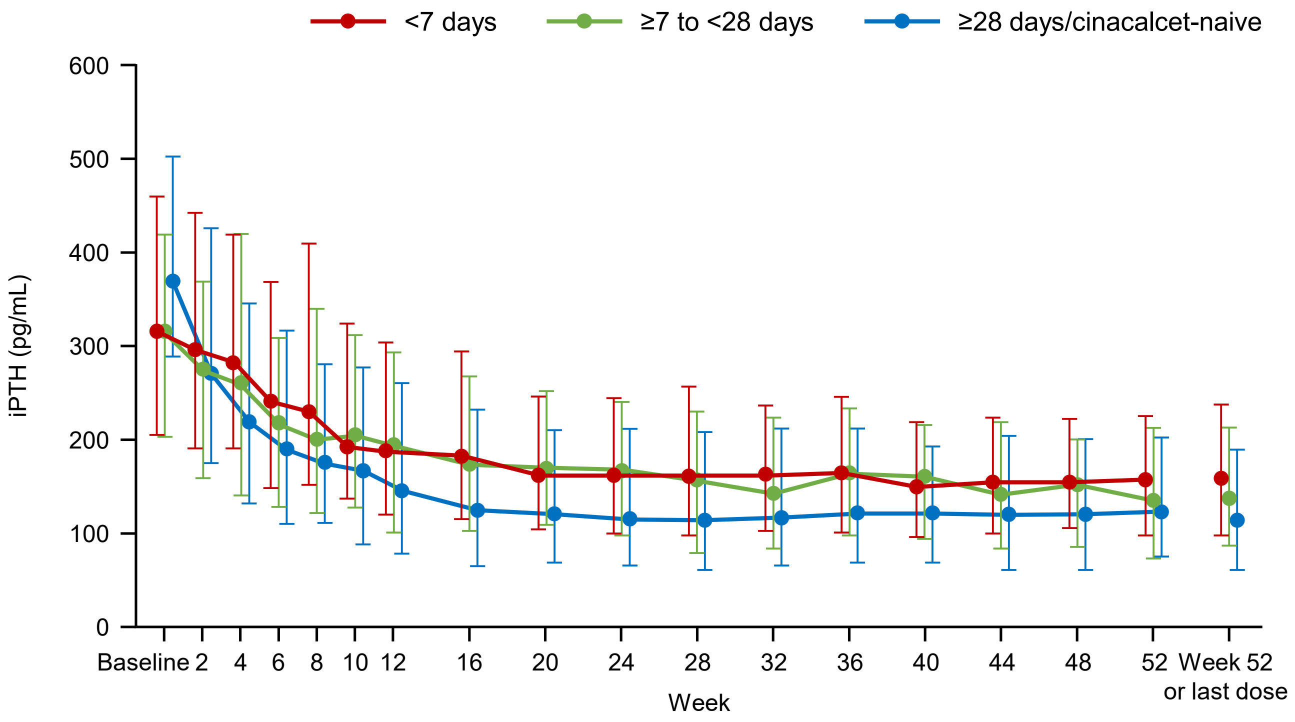

N	Baseline	2	4	6	8	10	12	16	20	24	28	32	36	40	44	48	52	Week 52 or last dose
<7 days	362	214	226	214	216	213	259	304	297	304	290	279	282	276	276	258	221	263
≥7 to <28 days	125	89	75	75	90	71	106	104	102	91	86	95	89	83	95	88	78	89
≥28 days /cinacalcet-naive	582	316	384	305	368	278	448	439	438	456	405	405	409	392	383	383	330	427

ESM Fig. 3 Changes in serum iPTH (a), cCa (b), P (c) and ALP (d) levels according to the time between the last dose of cinacalcet and the start of etelcalcetide

b. cCa

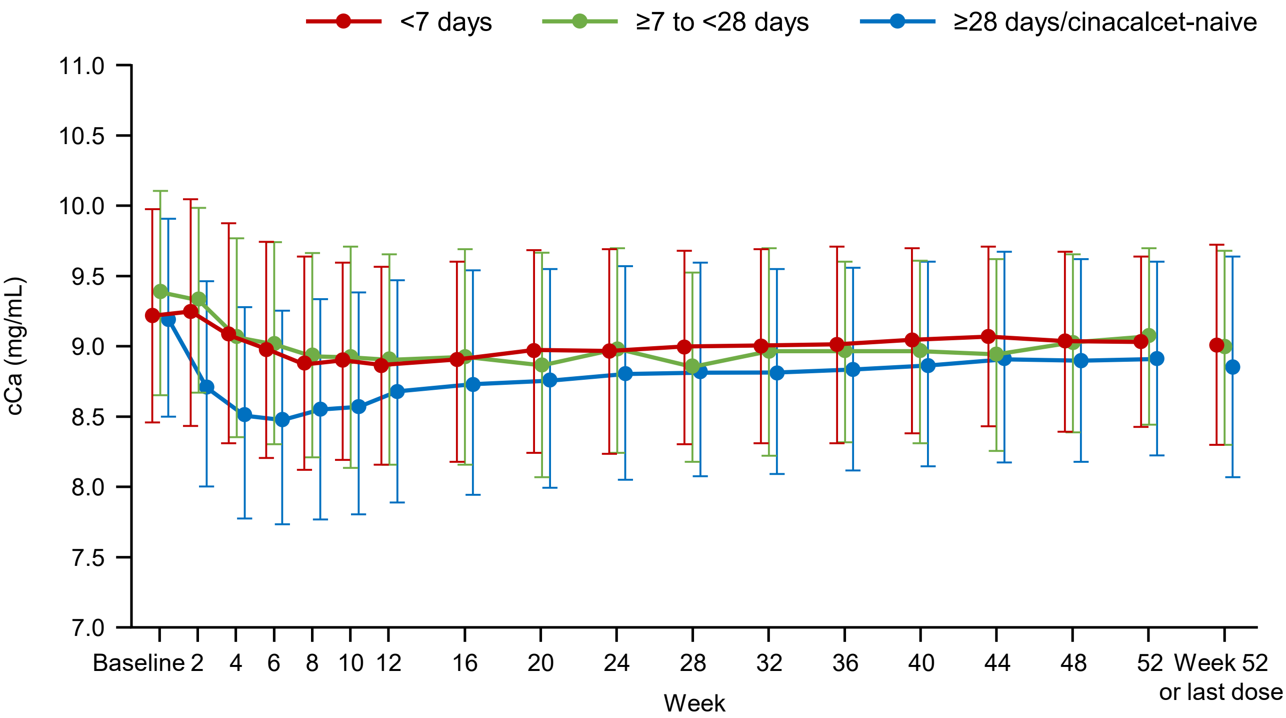

N	Baseline	2	4	6	8	10	12	16	20	24	28	32	36	40	44	48	52	Week 52 or last dose
<7 days	381	351	337	349	334	336	366	366	357	353	350	336	335	326	329	320	262	316
≥7 to <28 days	135	117	111	114	109	115	123	123	121	118	113	111	111	110	108	103	93	112
≥28 days /cinacalcet-naive	622	576	548	552	544	508	564	558	548	538	513	504	505	487	477	470	401	538

ESM Fig. 3 Changes in serum iPTH (a), cCa (b), P (c) and ALP (d) levels according to the time between the last dose of cinacalcet and the start of etelcalcetide

c. P

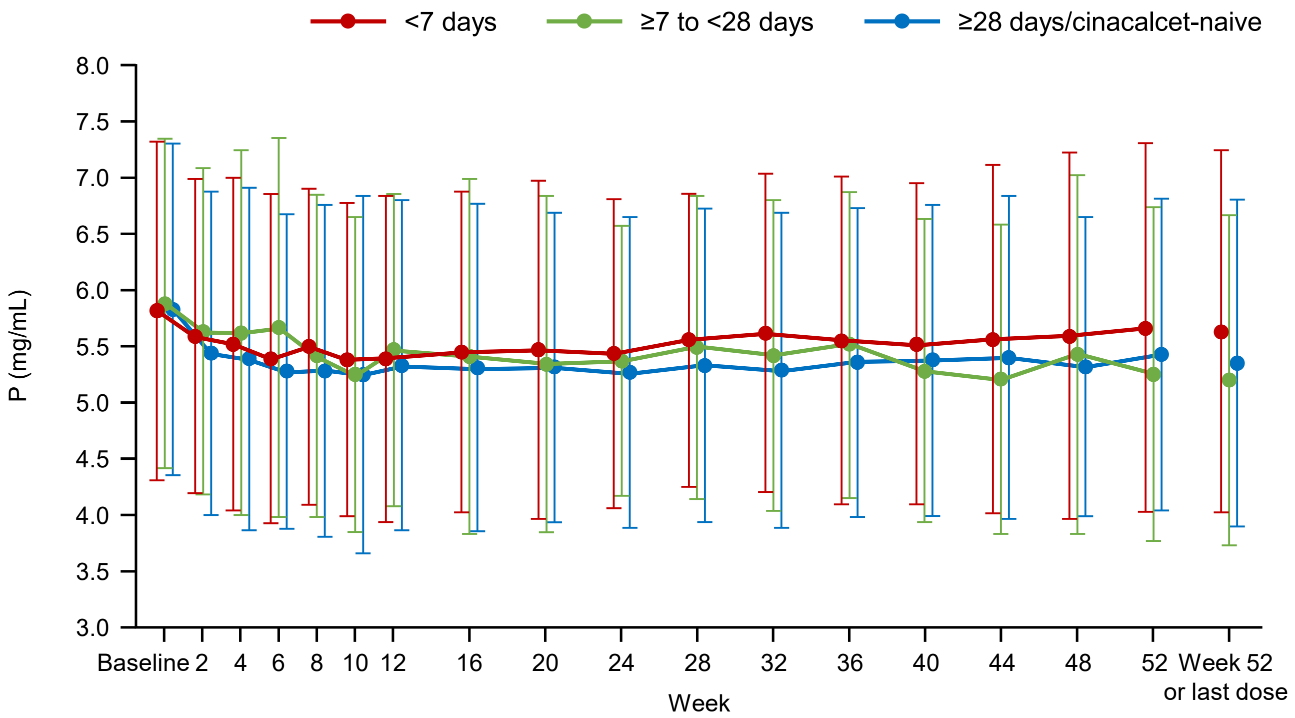

N	Baseline	2	4	6	8	10	12	16	20	24	28	32	36	40	44	48	52	Week 52 or last dose
<7 days	380	351	344	348	343	339	367	368	357	353	351	339	335	327	329	320	263	319
≥7 to <28 days	136	122	107	115	109	118	122	124	120	118	114	111	111	110	109	103	94	113
≥28 days /cinacalcet-naive	622	575	562	556	553	514	568	560	548	539	516	506	504	489	479	471	404	543

ESM Fig. 3 Changes in serum iPTH (a), cCa (b), P (c) and ALP (d) levels according to the time between the last dose of cinacalcet and the start of etelcalcetide

d. ALP

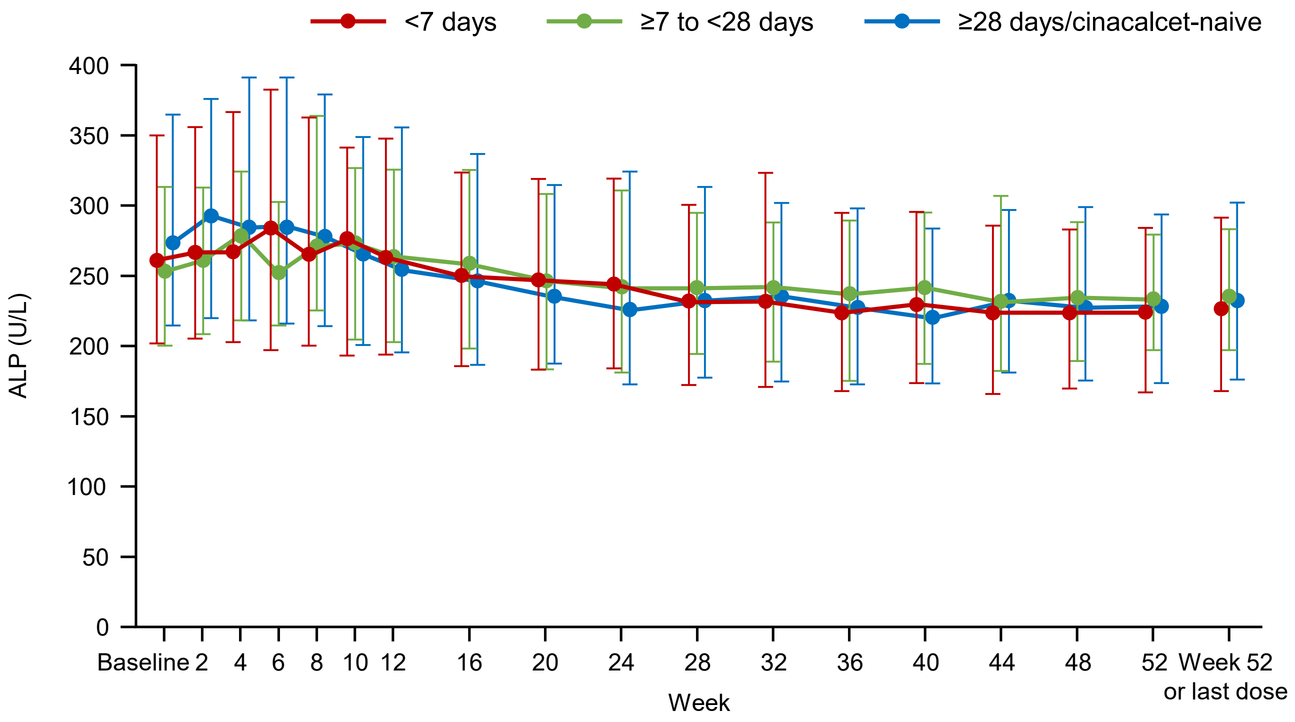

N																		
<7 days	336	199	212	194	223	185	266	306	304	298	294	278	277	283	272	267	218	255
≥7 to <28 days	118	64	62	61	64	63	80	92	96	92	87	97	94	90	88	85	71	78
≥28 days /cinacalcet-naive	508	281	336	268	332	245	406	441	427	430	410	409	407	397	379	372	301	379

ESM Fig. 4 Changes in serum iPTH (a), cCa (b), P (c) and ALP (d) levels according to baseline iPTH level

a. iPTH

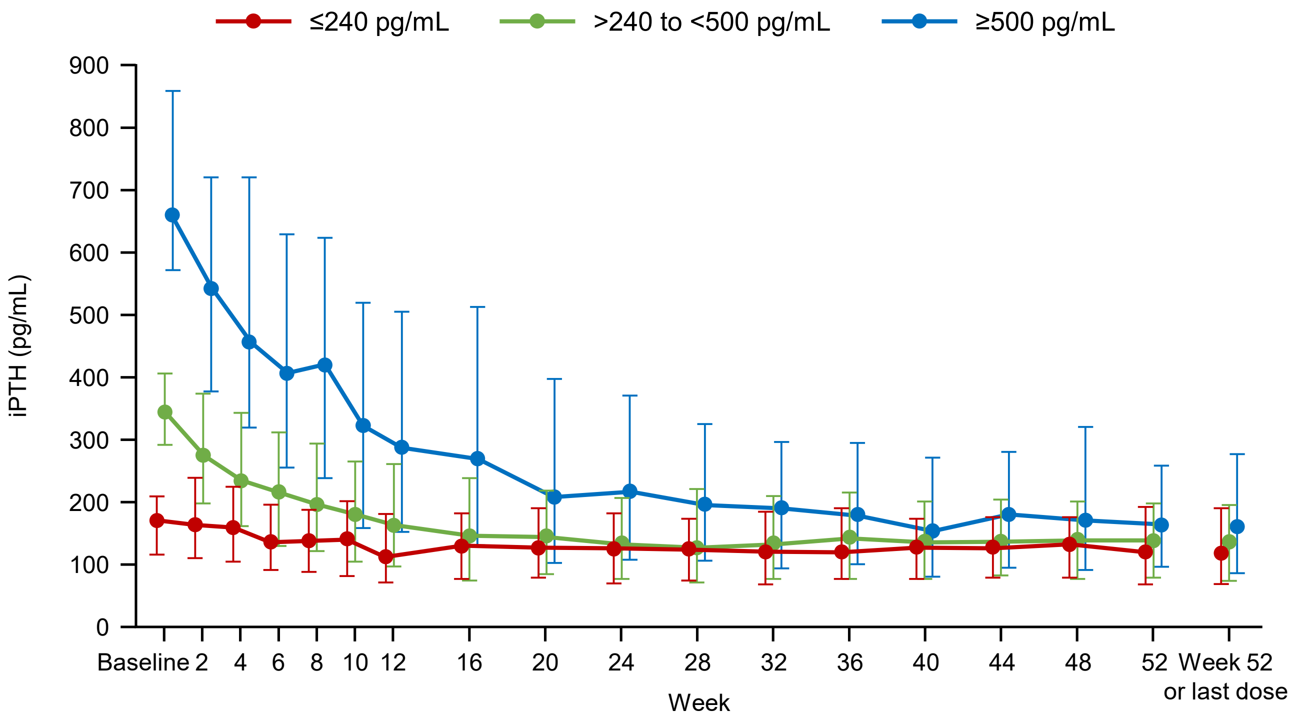

N																		
≤240	226	131	118	123	131	110	153	175	172	174	158	163	159	159	158	149	119	147
>240 to <500	618	326	388	322	385	292	435	442	439	455	401	405	393	393	381	382	341	428
≥500 pg/mL	240	119	133	117	127	118	173	175	169	179	174	165	182	156	166	154	138	159

ESM Fig. 4 Changes in serum iPTH (a), cCa (b), P (c) and ALP (d) levels according to baseline iPTH level

b. cCa

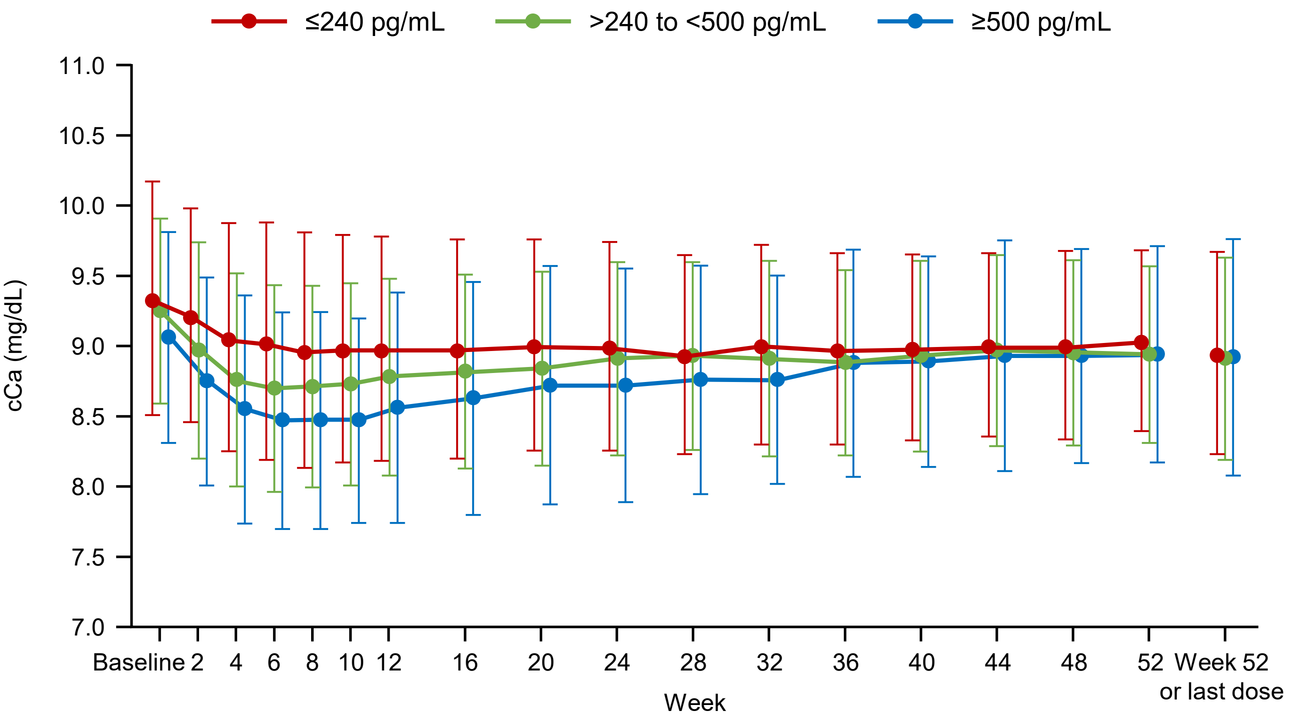

N	Baseline	2	4	6	8	10	12	16	20	24	28	32	36	40	44	48	52	Week 52 or last dose
≤240	225	204	182	200	192	181	203	205	201	197	187	186	187	182	179	173	147	182
>240 to <500	615	545	537	527	516	511	548	538	525	522	503	489	484	477	467	463	395	513
≥500	240	211	201	208	198	192	217	219	219	214	213	207	212	198	201	197	160	192
pg/mL																		

ESM Fig. 4 Changes in serum iPTH (a), cCa (b), P (c) and ALP (d) levels according to baseline iPTH level

c. P

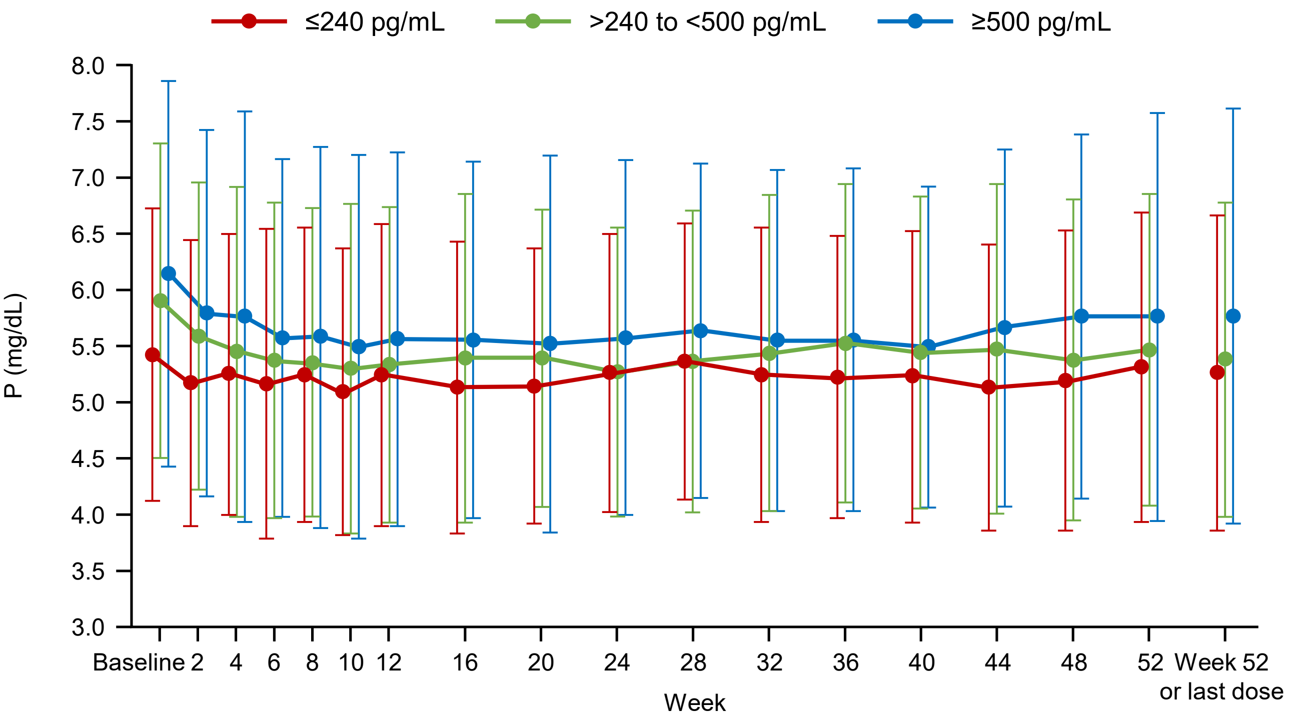

N																		
≤240	226	204	187	200	196	181	204	206	200	197	187	187	186	183	179	174	147	183
>240 to <500	617	553	553	536	531	524	553	542	528	523	508	493	485	479	471	464	397	518
≥500 pg/mL	240	213	201	208	198	196	218	221	218	215	214	208	212	199	201	196	161	194

ESM Fig. 4 Changes in serum iPTH (a), cCa (b), P (c) and ALP (d) levels according to baseline iPTH level

d. ALP

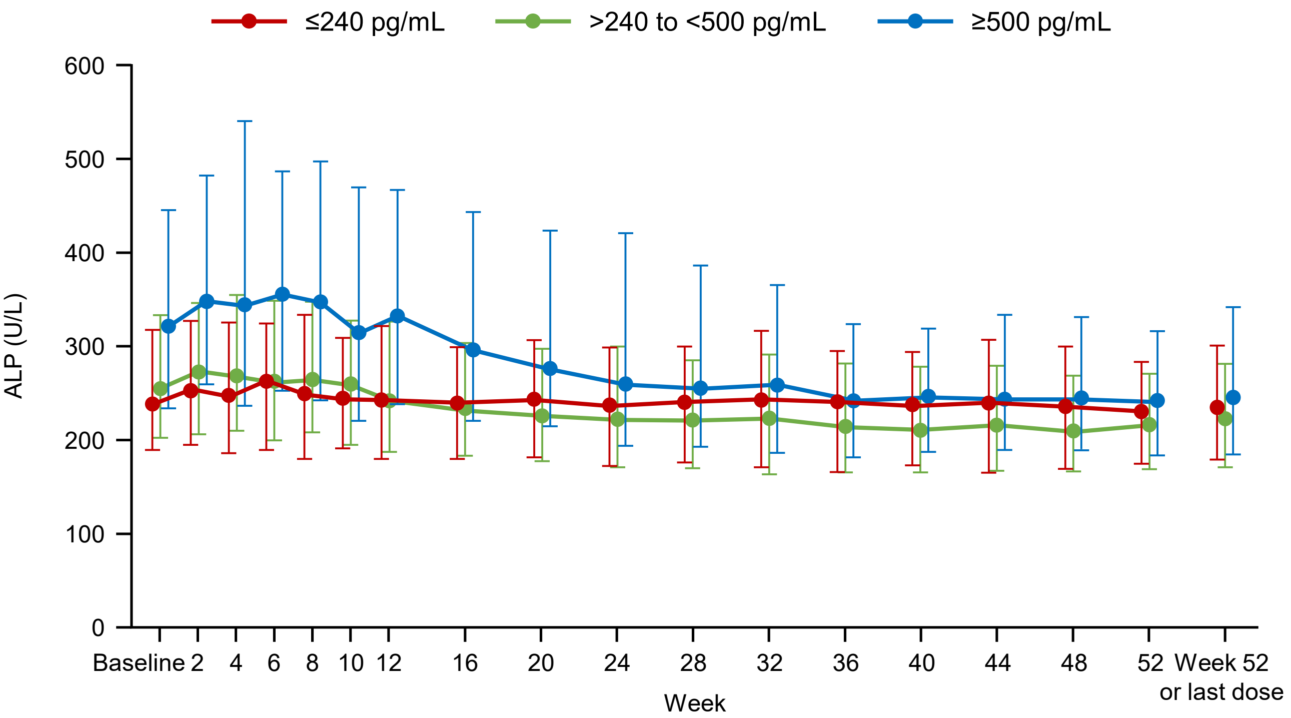

N	Baseline	2	4	6	8	10	12	16	20	24	28	32	36	40	44	48	52	Week 52 or last dose
≤240	187	124	111	123	117	115	131	168	163	160	151	160	156	160	152	142	105	123
>240 to <500	531	279	331	255	338	233	399	437	443	440	411	398	396	400	370	379	322	391
≥500 pg/mL	208	104	130	105	124	107	163	174	166	173	171	171	176	161	167	156	128	147

ESM Fig. 5 Changes in serum iPTH (a), cCa (b), P (c) and ALP (d) levels according to baseline Ca level

a. iPTH

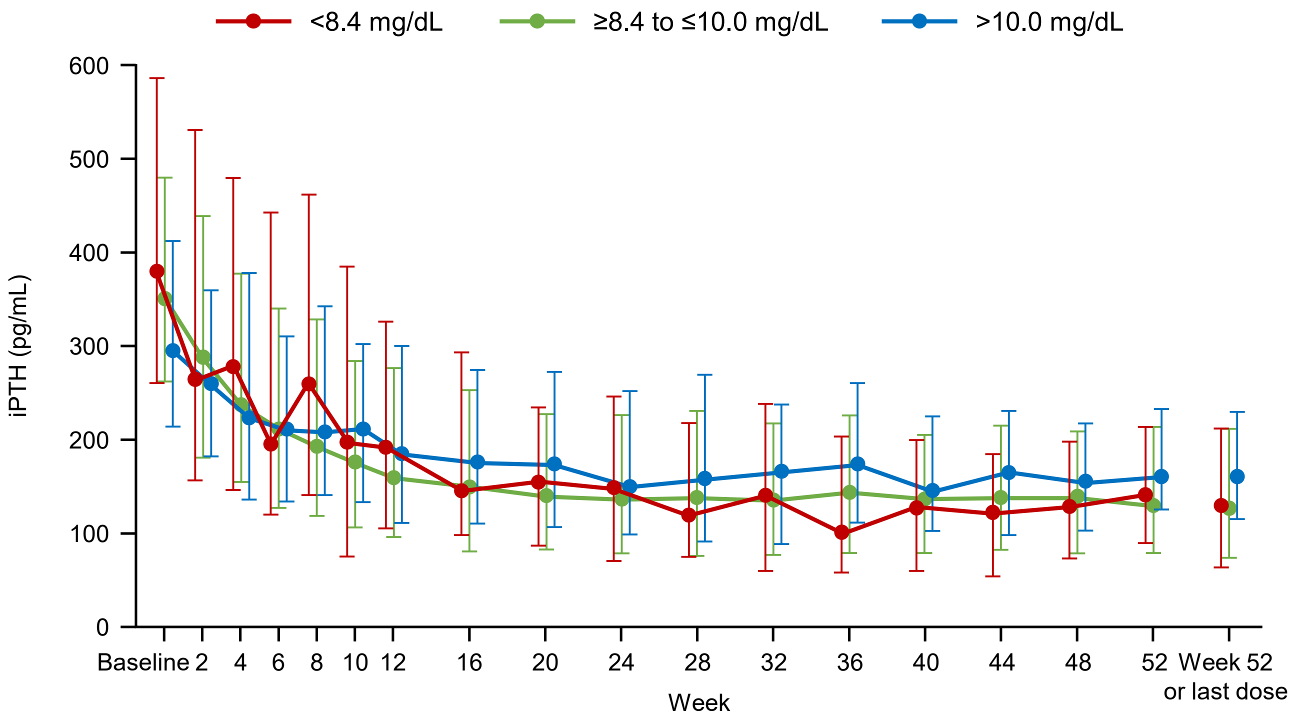

N	Baseline	2	4	6	8	10	12	16	20	24	28	32	36	40	44	48	52	Week 52 or last dose
<8.4	98	58	63	55	57	55	74	73	78	71	71	72	68	66	72	61	54	67
≥8.4 to ≤10.0	854	475	531	460	518	431	613	652	632	663	602	587	602	577	572	555	484	606
>10.0	128	80	76	75	89	64	104	105	112	104	97	103	92	94	94	95	75	88
pg/mL																		

ESM Fig. 5 Changes in serum iPTH (a), cCa (b), P (c) and ALP (d) levels according to baseline Ca level

b. cCa

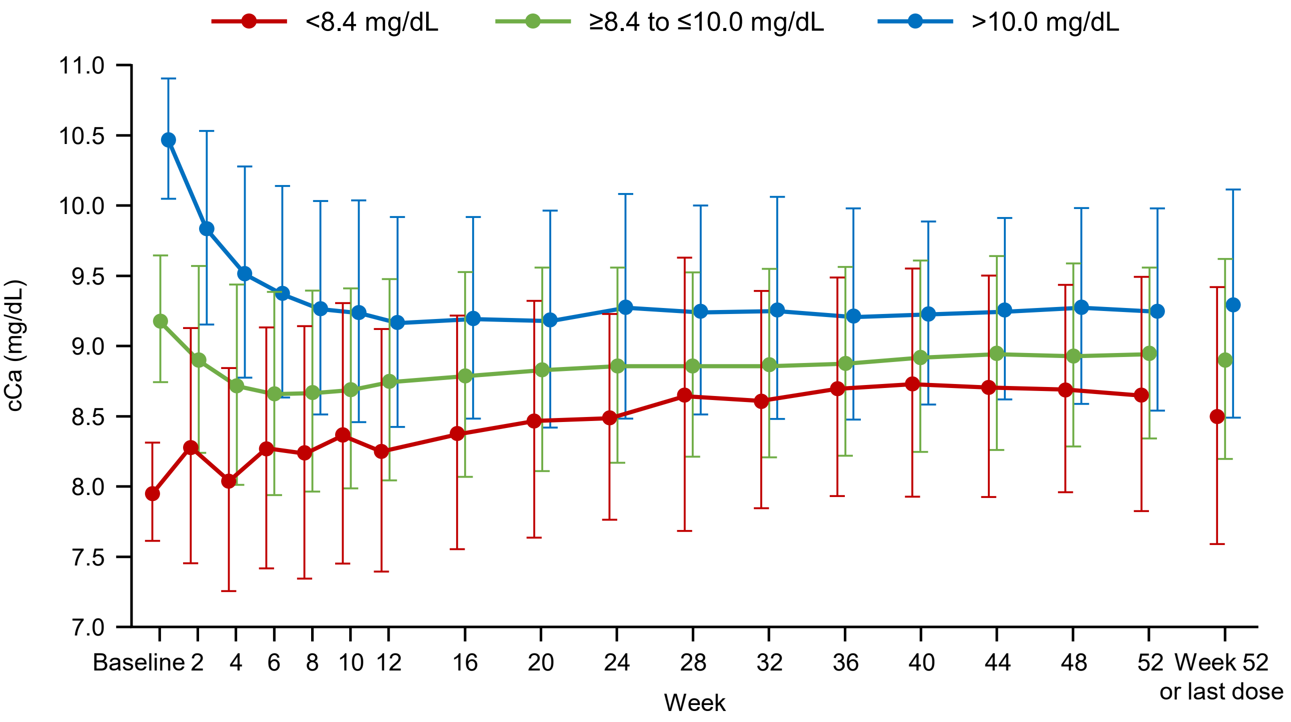

N	Baseline	2	4	6	8	10	12	16	20	24	28	32	36	40	44	48	52	Week 52 or last dose
<8.4	110	95	86	92	91	87	95	92	95	93	89	86	86	87	84	80	66	85
≥8.4 to ≤10.0	901	803	767	785	761	738	806	806	787	775	750	726	732	700	695	684	580	749
>10.0	142	128	124	117	118	114	128	129	125	124	121	121	115	118	118	112	95	113
pg/mL																		

ESM Fig. 5 Changes in serum iPTH (a), cCa (b), P (c) and ALP (d) levels according to baseline Ca level

c. P

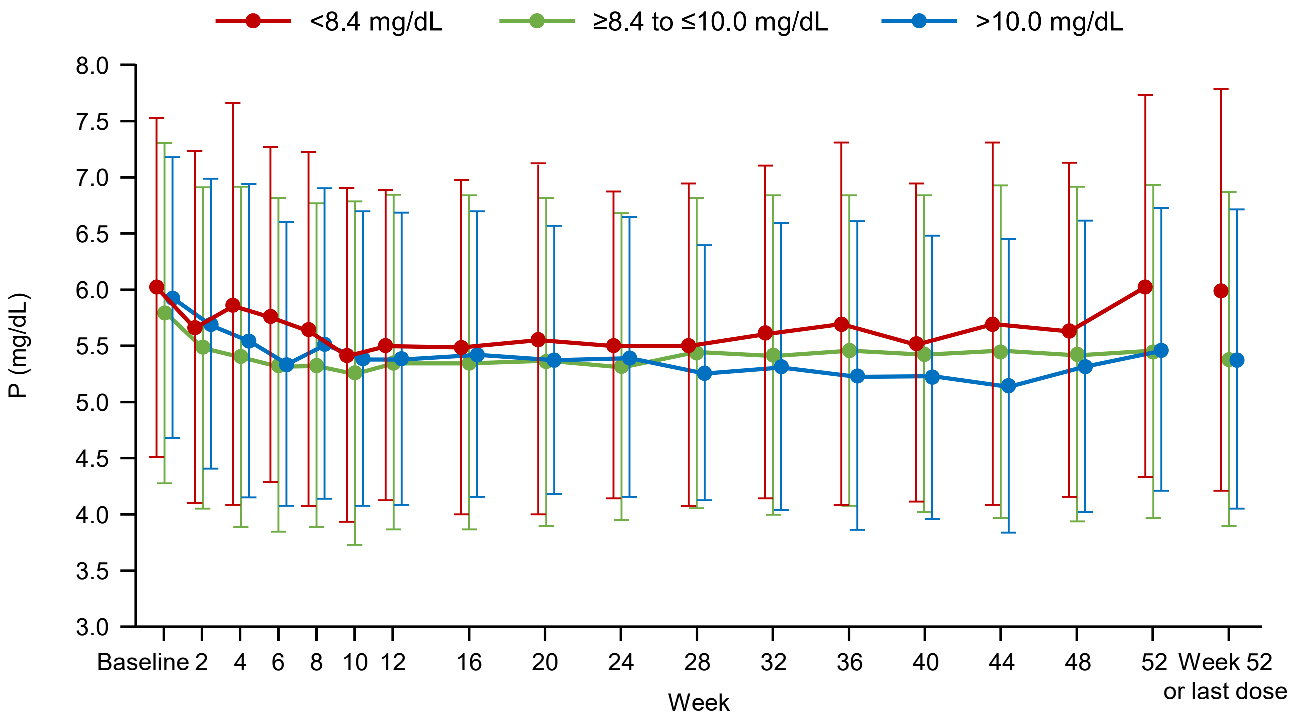

N	Baseline	2	4	6	8	10	12	16	20	24	28	32	36	40	44	48	52	Week 52 or last dose
<8.4	108	94	84	89	91	86	94	90	94	92	88	85	85	86	83	80	66	84
≥8.4 to ≤10.0	898	804	785	789	776	748	811	810	786	775	756	732	732	701	697	685	583	754
>10.0	141	129	124	118	119	115	127	130	125	125	121	120	114	120	119	111	96	116
pg/mL																		

ESM Fig. 5 Changes in serum iPTH (a), cCa (b), P (c) and ALP (d) levels according to baseline Ca level

d. ALP

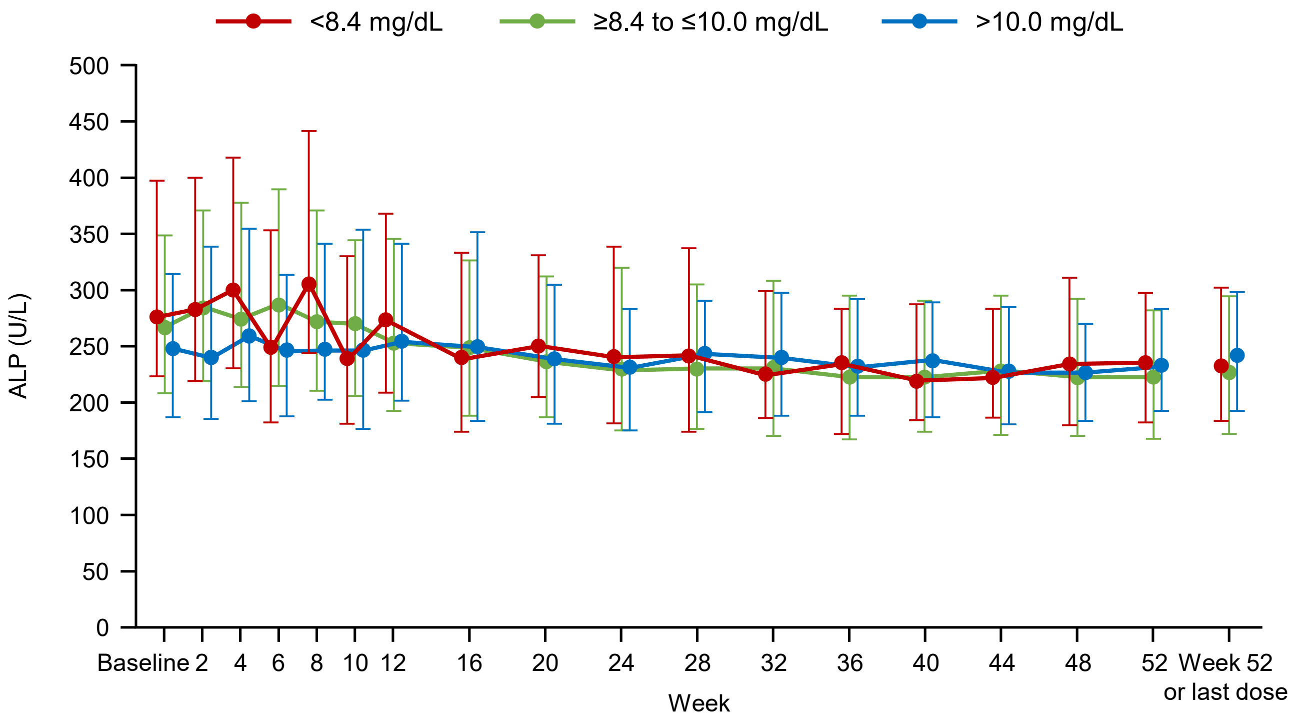

N																		
<8.4	98	50	61	49	64	43	74	76	80	72	75	73	73	74	74	66	52	66
≥8.4 to ≤10.0	762	418	472	403	471	382	571	643	636	636	607	602	599	591	558	560	450	543
>10.0	110	66	65	58	73	54	88	102	97	99	96	91	88	91	90	84	70	81
pg/mL																		
